# Decoupling H‑Release and OH^–^ Management at Pd@TiO_2_ Interfaces for Efficient Alkaline Hydrogen Oxidation Reaction

**DOI:** 10.1021/acscatal.5c08285

**Published:** 2026-02-12

**Authors:** Benjin Jin, Antti-Jussi Kallio, Nils Rieger, Vasyl Marchuk, Cedric Schiwek, Junjie Shi, Jani Sainio, Hua Jiang, Amine Hammouali, Jefina A. S. Koivuniemi, Nana Han, Björn Wickman, Simo Huotari, Tanja Kallio

**Affiliations:** † Department of Chemistry and Materials Science, School of Chemical Engineering, 174277 Aalto University, P.O. Box 16100, Aalto, Espoo FI-00076, Finland; ‡ Department of Physics, 3835 University of Helsinki, P.O. Box 64, Helsinki FI-00014, Finland; § Department of Physics and Competence Centre for Catalysis, 11248 Chalmers University of Technology, Göteborg SE-412 96, Sweden; ∥ 150232 ESRF, The European Synchrotron, Grenoble 38043, France; ⊥ Department of Applied Physics, School of Science, Aalto University, P.O. Box 15100, Aalto, Espoo FI-00076, Finland

**Keywords:** operando X-ray absorption spectroscopy, identical location TEM, core−shell catalysts, hydrogen oxidation reaction, fuel cell

## Abstract

Operando-level insight into catalyst degradation and reaction mechanisms is essential for progress in the alkaline hydrogen oxidation reaction (HOR). Herein, these aspects are investigated using a core–shell Pd@TiO_2_/C catalyst synthesized by thermal reduction followed by atomic layer deposition. The obtained catalyst exhibits high stability and delivers a mass exchange current density (*j* ^0,m^) of 97.5 mA mg_Pd_^–1^, more than three times that of uncoated Pd/C (27.5 mA mg_Pd_^–1^). Identical location transmission electron microscopy reveals a growth–detachment degradation pathway for Pd/C during accelerated durability testing, whereas the TiO_2_ shell in Pd@TiO_2_/C effectively suppresses this degradation, resulting in enhanced structural stability. Operando X-ray absorption spectroscopy under device-relevant conditions demonstrates the complementary functions of the two components: hydrogen dissociates and forms PdH_*x*_ on the Pd core, lowering its Fermi level and driving electron transfer from TiO_2_ to Pd, while the TiO_2_ shell facilitates hydrogen desorption and provides OH^–^ adsorption sites, thereby accelerating the reaction kinetics. These findings elucidate the dual stabilizing and catalytic roles of TiO_2_ and suggest a promising strategy for the design of durable and efficient alkaline HOR catalysts.

## Graphical abstract

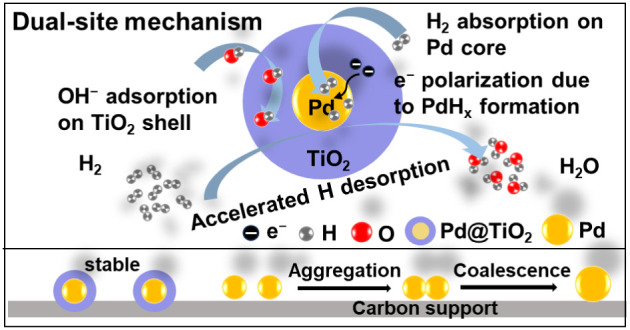

## Introduction

Alkaline anion exchange membrane fuel cells (AEMFCs) have gained increasing attention owing to advances of anion exchange membranes, which enable reduced costs and greater operational flexibility compared to proton exchange membrane fuel cells (PEMFCs).^1–3^ However, the sluggish kinetics of the hydrogen oxidation reaction (HOR) in alkaline media remains a major challenge.^4^ Even with platinum-based catalysts, the HOR kinetics in alkaline media are several orders of magnitude slower than in acidic conditions, severely limiting overall fuel cell performance.^5^

To overcome this limitation, extensive efforts have focused on engineering Pd-based electrocatalysts for efficient HOR in alkaline media owing to their electrocatalytic properties comparable to those of Pt.^6–10^ One promising strategy is coupling Pd with metal oxides, which has been shown to markedly enhance catalytic activity.^11,12^ Among various oxides, TiO_2_ is particularly attractive because of its ability to induce strong metal–support interactions (SMSIs) that alter the electronic structure of supported metals.^13^ Gorodetskii et al. demonstrated that in the Pd–Ti^3+^/TiO_2_ system, hydrogen atoms dissociated on Pd can transport to the Ti^3+^ defect sites of TiO_2_ via a spillover mechanism, where the hydrogen oxidation reaction then proceeds on both the Pd surface and the reduced oxide support.^14^ More recently, Tang et al. revealed that oxygen vacancies in TiO_2_ not only facilitate hydrogen spillover from Pd nanoparticles but also enable reversible electron transfer between Pd and Ti, dynamically modulating the Ti^3+^/Ti^4+^ states and accelerating hydrogen desorption during formate oxidation.^15^ Although hydrogen spillover on Pd–TiO_2_ interfaces has been extensively investigated, the specific impact of TiO_2_-induced SMSI on the HOR in alkaline media remains insufficiently understood.^16^

Moreover, the mechanistic understanding of Pd-based catalysts for alkaline HOR remains limited, largely owing to the scarcity of operando evidence under device-relevant conditions. The intrinsic ability of Pd to absorb hydrogen into its lattice further complicates interpretation by introducing additional states and interactions beyond simple surface adsorption.^17^ While in situ characterizations using rotating disk electrode (RDE) setups can yield valuable insights, they often fail to replicate the complex environment of a working membrane electrode assembly. In contrast, operando techniques provide a more representative view of catalytic behavior under actual operating conditions.^18^ However, such studies remain scarce because of significant technical challenges associated with operando measurements in electrochemical systems, including complex cell design, limited gas flow and temperature control, and setup stability issues. To date, direct operando observation of Pd–H interactions in functional AEMFCs remains elusive.

Apart from kinetic considerations, the stability of Pd-based catalysts is a critical concern for their HOR performance. Catalyst degradation is a multifaceted process influenced by operating conditions, such as voltage fluctuations, gas flow, and temperature.^19,20^ A comprehensive understanding of degradation mechanisms is essential for the rational design of durable materials with prolonged operational lifetimes.^21,22^ Identical location transmission electron microscopy (IL-TEM) has emerged as a powerful technique for elucidating degradation pathways by enabling direct visualization of morphological changes at the same catalyst location before and after operation.^23,24^ This technique allows the identification of specific degradation modes, such as particle agglomeration, detachment, and structural deformation, providing valuable insights into catalyst failure mechanisms.

In this work, a core–shell structured Pd@TiO_2_/C catalyst was prepared via thermal reduction followed by atomic layer deposition, serving as a model system to investigate degradation pathways and reaction mechanisms in the alkaline HOR. To correlate catalytic performance with structural evolution and to elucidate TiO_2_-induced interfacial effects, operando X-ray absorption spectroscopy (XAS) and IL-TEM are employed. This combined approach aims to clarify how the metal–oxide interface governs hydrogen adsorption and catalyst degradation, thereby providing mechanistic insight and design principles for developing durable alkaline HOR catalysts.

## Methods

### Reagents and Materials

Potassium hydroxide (KOH, 85%, extra pure, flakes), Palladium­(II) chloride (PdCl_2_, 99.99%), and isopropanol (C_3_H_8_O, 99.5+%) were purchased from Thermo Scientific. Platinum on activated carbon powder (20 wt % Pt) and hydrochloric acid (34 wt %) were obtained from Alfa Aesar. Titanium­(IV) isopropoxide (TTIP, 97%) was purchased from STREM Chemicals. Ethanol (99.5%) was purchased from the Anora Group. Vulcan XC-72R (GP-3875) was purchased from Cabot Corporation. Nafion 117 (5 wt %) was purchased from Sigma-Aldrich. Anion exchange membranes, including Sustainion X37–50 and Fumasep FAA-3–50, as well as gas diffusion layers (Sigracet 22 BB), were purchased from Fuel Cell Store. PiperION A80-HCO_3_ membranes were obtained from Fuel Cell Earth. Deionized water (Synergy, resistivity: 18 MΩ/cm at 25 °C) was used in all procedures. All chemicals were used as received without further purification unless otherwise stated.

### Synthesis of Pd/C and Pd@TiO_2_/C

In a typical procedure, approximately 80 mg of PdCl_2_ was dissolved in 20 mL of ethanol under constant stirring. Concentrated hydrochloric acid was added dropwise until a transparent reddish solution was obtained. This solution was diluted to 25 mL by using a volumetric flask to prepare the Pd precursor solution. Separately, about 20 mg of Vulcan XC 72R carbon (VC) was treated with ozone (ozone generator, OZX-300ST, maximum power) for 40 min in a fume hood to introduce surface oxygen functionalities. The ozone-treated VC was subsequently dispersed in ethanol (10 mg mL^–1^) and stirred vigorously (magnetic stirrer, Heidolph MR Hei-Tec, 1000 rpm). The required volume of the Pd precursor solution was added dropwise under continuous stirring to achieve the desired Pd loading. The resulting ink was stirred for 2 days to ensure complete adsorption and homogenization. Afterward, the mixture was dried at 80 °C in a thermostatic oven (Fratelli Galli G-Cell). The dried powder was transferred to a tube furnace (Nabertherm RS80/500/11), flushed with nitrogen for 30 min, and then heated at a rate of 200 °C h^–1^ to 200 °C under N_2_. Once at 200 °C, the sample was maintained for 2 h under a reducing atmosphere of 5% H_2_ in Ar. After cooling to room temperature under a N_2_ flow, the Pd/C catalyst was obtained. A flow-type hot-wall atomic layer deposition (ALD) reactor (F-120, ASM Microchemistry Ltd.) was used for TiO_2_ deposition. The Pd/C powder was used as the substrate and loaded into a dedicated powder chamber within the reactor. To initiate the deposition process, 1 mL of TTIP was placed in a glass boat positioned inside the precursor chamber, maintained at a temperature between 70 and 140 °C. The deposition chamber was heated to 220 °C and purged with high-purity nitrogen for 50 min with a flow speed of 200 ccm to ensure a clean and inert environment. Each ALD cycle consisted of the following sequence: TTIP pulse (10 s), N_2_ purge (20 s), H_2_O pulse (10 s), and N_2_ purge (30 s). This cycle was repeated for a defined number of times to prepare Pd@TiO_2_/C samples.

### Characterization

TEM images and high-angle annular dark-field scanning transmission electron microscopy (HAADF-STEM) images of all samples were recorded on a JEOL JEM-2800 (Analytical HRTEM) at 200 kV. Atomic resolution HAADF-STEM was taken using a JEOL2200FS, Cs-corrected HRTEM at an acceleration voltage of 200 kV. XRD patterns were recorded on a PANalytical X’Pert PRO diffractometer using Cu Kα radiation (λ = 1.5406 Å) operated at 40 kV and 40 mA. Data were collected over a 2θ range of 10–90° with a step size of 0.025°. The samples were mounted as thin layers on a white gasket. Phase identification was performed by using the ICDD database (JCPDS No. 46-1043 for fcc-Pd). XPS was carried out with a Kratos Axis Ultra spectrometer with monochromated Al K_α_-radiation, a pass energy of 40 eV, an X-ray power of 150 W, and an analysis area of approximately 700 μm x 300 μm.

### HOR Performance Evaluation

The catalyst ink was prepared by dispersing 4 mg of catalyst in 1000 μL of solution composed of 970 μL of isopropyl alcohol and 30 μL of Nafion solution. The mixture was ultrasonicated for 20 min to ensure a homogeneous dispersion. A total of 8 μL of the well-dispersed ink was drop-cast onto a glassy carbon electrode and allowed to dry in ambient air. An additional 8 μL of ink was then applied, resulting in a total catalyst loading of 326 μg_cat_ cm^–2^. Prior to ink deposition, the GC electrode tip was polished using a 0.05 μm alumina slurry, followed by sonication in a mixture of ultrapure water and ethanol for 3 min.

The hydrogen oxidation reaction measurements were carried out in a standard three-electrode cell (Pine Research Instrumentation, made of borosilicate glass) in 0.1 M KOH by using an Autolab potentiostat (PGSTAT128N, Metrohm Autolab B.V.). The working electrode was a glassy carbon disk modified with the electrocatalyst. A hydrogen electrode (ET070, eDAQ Instrument) and a graphite rod (6 mm diameter) served as the reference and counter electrodes, respectively. All experiments were conducted at room temperature (25 °C), and all measured potentials in linear sweep voltammetry (LSV) were referenced to the reversible hydrogen electrode (RHE) with 100% *i*R compensation.

Initially, cyclic voltammetry (CV) was performed in the potential range of 0.005 to 0.4 V vs RHE at a scan rate of 100 mV s^–1^ until overlapping curves were obtained. Subsequently, CO stripping was employed to clean the catalyst surface. After the electrolyte was saturated with H_2_, LSV was conducted at a scan rate of 5 mV s^–1^ under a rotating speed of 1600 rpm. Electrochemical impedance spectroscopy (EIS) was performed in the frequency range of 100 kHz to 0.1 kHz with an AC amplitude of 8 mV.

The electrochemically active surface area (ECSA) was determined via CO stripping voltammetry, which provides the electrochemically accessible Pd surface area. CO adsorption was achieved by holding the electrode at 0.1 V in CO-saturated 0.1 M KOH for 10 min, followed by purging with N_2_ for 30 min to remove excess CO. CO stripping was then performed from 0.1 to 1.2 V vs RHE at a sweep rate of 20 mV s^–1^. The ECSA was calculated by the following equation:

$$ECSA_{CO}=\frac{Q_{CO}}{Q_{s}}$$

where *Q*_CO_ is the measured integrated charge of the CO oxidation peak and *Q*_s_ is the surface charge density of 420 μC cm^–2^ assumed for monolayer CO adsorption on a Pd metal.

The kinetic current density (*j* ^k^) can be deduced using the Koutechy–Levich equation:

$$\frac{1}{j}=\frac{1}{j^{k}}+\frac{1}{j^{d}}=\frac{1}{j^{k}}+\frac{1}{Bc_{0}\omega^{1/2}}$$

where *j* is the measured current density, *B* is the Levich constant, *c*_0_ is the solubility (7.33 × 10^–4^ mol L^–1^) of H_2_, and ω is the rotation speed of the electrode.

The exchange current density (*j* ^0^) can be extracted from the Butler–Volmer equation:

$$j^{k}=j^{0}\left[e^{\frac{\alpha F}{RT}\eta }-e^{\frac{-\left(1-\alpha \right)F}{RT}\eta }\right]$$

where α is the charge transfer coefficient, η is the overpotential, *R* is the universal gas constant (8.314 J mol^–1^ K^–1^), *T* is the temperature (303 K), and *F* is the Faraday constant.

Nonlinear fitting was performed using OriginPro 2025 and was applied in the overpotential range of ±20 mV, chosen to minimize noise near equilibrium and avoid mass-transport limitations. α was not fixed during the fitting.

The stability of the catalysts was evaluated through an accelerated durability test (ADT) conducted in H_2_-saturated 0.1 M KOH. Prior to the ADT protocol, LSV was performed at a scan rate of 5 mV s^–1^ to assess the initial HOR performance. The ADT was carried out by cycling the potential between 0 and 0.4 V vs RHE for 7000 cycles at a scan rate of 100 mV s^–1^. Following the durability cycling, two CV scans were performed between 0.05 and 1.2 V vs RHE at 20 mV s^–1^ to remove surface contaminants. A final LSV measurement was then conducted under the same conditions as the initial scan to evaluate performance degradation. The electrolyte was purged with H_2_ throughout the test to avoid interference from the dissolved gases.

## Results and Discussion

### Physical Characterization

The Pd@TiO_2_/C was synthesized by depositing TiO_2_ onto the Pd/C sample using ALD. Briefly, first, Pd/C was prepared by thermal reduction by mixing a concentrated PdCl_2_ solution with O_3_-treated Vulcan carbon in ethanol, followed by stirring for 72 h. After solvent evaporation, the mixture was thermally treated at 200 °C for 2 h under a reducing atmosphere (5% H_2_ in Ar). The product was cooled to room temperature under N_2_ to yield Pd nanoparticles on carbon. Transmission electron microscopy (TEM) images in Supporting Information (Figure S1) confirm that the Pd nanoparticles are uniformly dispersed on the carbon support. TiO_2_ was subsequently deposited onto Pd/C by ALD, with the loading controlled by adjusting the number of deposition cycles. This process produced Pd nanoparticles on Vulcan carbon with a porous TiO_2_ overlayer at low deposition cycles. Based on its performance in hydrogen oxidation reaction (HOR) experiments (detailed discussion below in the section “Electrochemical Characterization”), the Pd@TiO_2_/C sample prepared with 24 ALD cycles was selected as the optimized catalyst for detailed physicochemical characterization.

Morphological and structural characteristics of the Pd@TiO_2_/C catalyst are presented in Figure 1. High-angle annular dark-field scanning transmission electron microscopy (HAADF-STEM) in Figure 1a and TEM images in Figure 1b show a uniform distribution of nanoparticles. Higher magnification images in Figure 1c and the bright-field TEM image in Figure 1d further confirm the core–shell architecture, as evidenced by contrast differences. At the ALD cycle numbers employed here, TiO_2_ does not form a fully closed, pinhole-free shell on Pd. Instead, the coating is porous with some variation in thickness, as seen in Figure 1c. Elemental mapping via energy-dispersive X-ray spectroscopy (STEM-EDS) in Figure 1e shows well-defined Pd nanoparticles, while Ti is uniformly distributed across the carbon support. This distribution suggests that the Ti precursors interact with the Pd surface and also anchor directly onto carbon, likely due to the presence of oxygen-containing functional groups introduced during ozone treatment. The EDS line-scan profile in Figure S2 suggests that TiO_2_ growth is strongly favored at the Pd surface under our deposition conditions. X-ray diffraction (XRD) (Figure S3) shows reflections at 2θ = 40.02°, 46.49°, 68.05°, 81.74°, and 86.24°, corresponding to the (111), (200), (220), (311), and (222) planes of face-centered cubic (fcc) Pd (JCPDS No. 87-0641, space group Fm3̅m). No discernible peaks attributable to crystalline TiO_2_ are observed, suggesting that the TiO_2_ shell is poorly crystalline.

**Figure 1:**
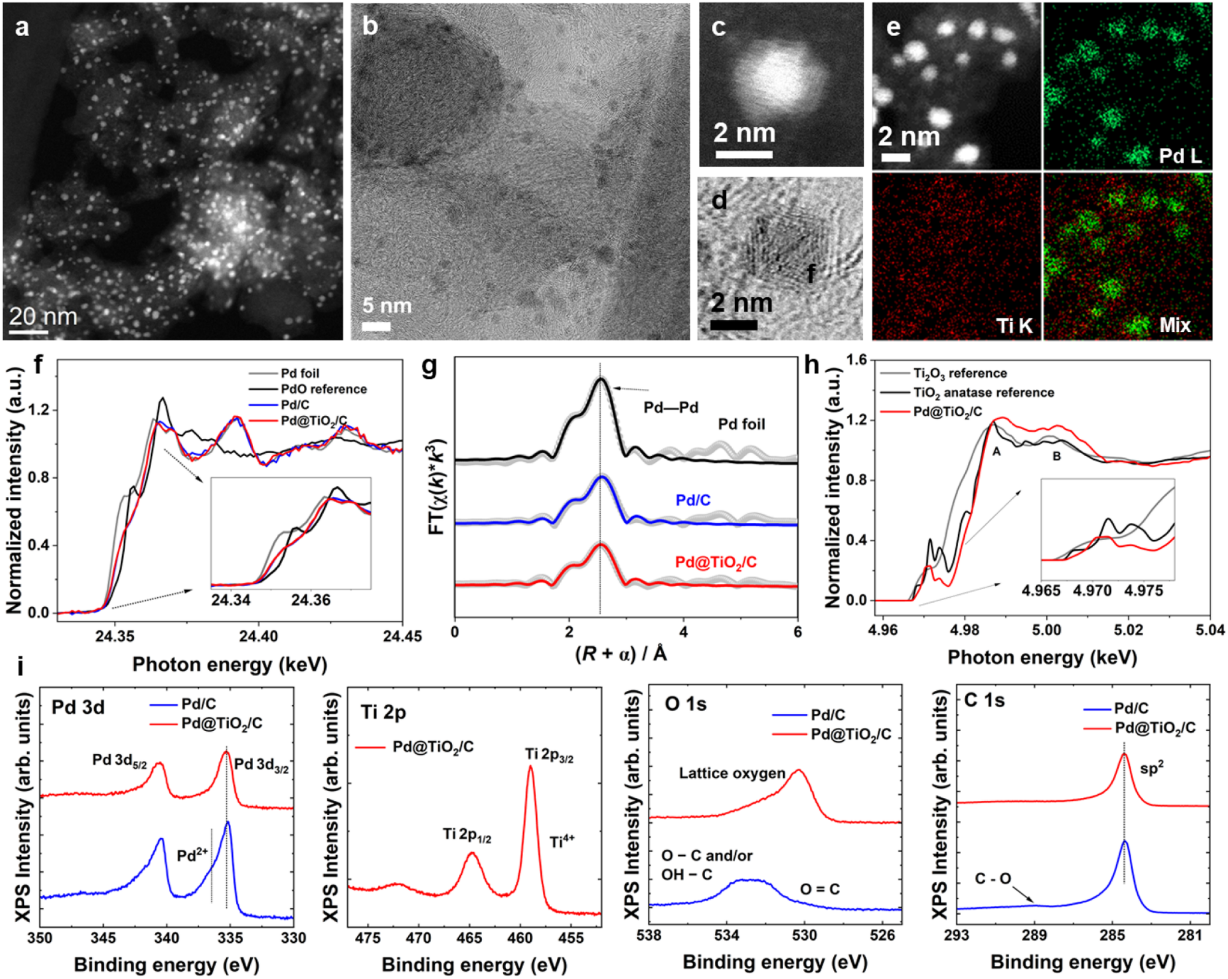
Structural and electronic characterization of Pd@TiO_2_/C. **a)** HAADF-STEM image, **b)** TEM image, **c)** an enlarged HAADF-STEM image, and **d)** HR-TEM image of a Pd@TiO_2_/C sample. **e)** HAADF-STEM image and corresponding EDS elemental maps of Pd and Ti in Pd@TiO_2_/C. **f)** Normalized Pd K-edge XANES spectra of a Pd foil, PdO, Pd/C, and Pd@TiO_2_/C. **g)** Fourier-transformed EXAFS spectra and corresponding fits for Pd foil, Pd/C, and Pd@TiO_2_/C. **h)** Normalized Ti K-edge XANES spectra of TiO_2_, Ti_2_O_3_, and Pd@TiO_2_/C. **i)** XPS spectra of the Pd 3d, Ti 2p, O 1s, and C 1s regions for Pd/C and Pd@TiO_2_/C.

To probe the local electronic structure and oxidation states, X-ray absorption spectroscopy (XAS) was collected. The Pd K-edge X-ray absorption near edge (XANES) spectra of Pd foil, PdO reference, Pd/C, and Pd@TiO_2_/C are shown in Figure 1f. The Pd foil exhibits a sharp absorption edge at ∼24.353 keV and a relatively weak white line characteristic of metallic Pd^0^. In contrast, the PdO reference displays a positively shifted edge and an intense white line indicative of Pd^2+^. Both Pd/C and Pd@TiO_2_/C exhibit absorption edges located between those of Pd foil and PdO. All spectra exhibit a shoulder near 24.355 keV (see the inset in Figure 1f), which is sharp in the Pd foil and PdO reference but less defined in the Pd/C and Pd@TiO_2_/C. This broadening is likely due to lattice disorder or nanoscale effects in the catalyst. The Fourier-transformed extended X-ray absorption fine structure (EXAFS) spectra of Pd/C and Pd@TiO_2_/C (Figure 1g) exhibit a dominant peak at ∼2.7 Å, corresponding to the first-shell Pd–Pd coordination. The coordination numbers (*N*) are 8.7 for Pd/C and 8.0 for Pd@TiO_2_/C, which are lower than those for the Pd foil (*N* = 12), consistent with the small particle size of the catalyst (Figure S4 and Table S1). TiO_2_ prepared by ALD is known to contain intrinsic defects, including oxygen vacancies and reduced Ti^3+^ species.^25,26^ The Ti K-edge XANES spectra in Figure 1h provide insight into the titanium oxidation states. The rising edge of Pd@TiO_2_/C closely matches that of the TiO_2_ anatase reference, indicating that Ti predominantly exists in the Ti^4+^ state.^27^ Weak pre-edge features between 4.965 and 4.980 keV, attributed to 1s → 3d transitions enabled by Ti 3d–O 2p hybridization, are consistent with a distorted octahedral environment typical of anatase or rutile.^28,29^ The slightly reduced intensity of these pre-edge features in Pd@TiO_2_/C suggests a more symmetric local environment, possibly caused by interfacial electronic interactions between Pd and TiO_2_.^29^ Compared with the rutile TiO_2_ reference, the Ti K-edge XANES spectrum of Pd@TiO_2_/C more closely resembles that of anatase TiO_2_ (Figure S5). This assignment is supported by the closer match of both A and B peak positions and relative intensities to those of the anatase reference.

X-ray photoelectron spectroscopy (XPS) in Figure 1i was used to further investigate the surface chemical states. The survey spectra indicate that the samples are free from impurities and contain only the expected elements (Figure S6). In the Pd 3d region, Pd/C shows two components: a main Pd^0^ peak at ∼335.2 eV (3d_5/2_) and a shoulder at 337–338 eV, corresponding to Pd^2+^ species (likely PdO).^30,31^ In contrast, Pd@TiO_2_/C shows predominantly metallic Pd, with the shoulder peak largely suppressed, indicating a reduced level of surface oxidation after TiO_2_ coating. Pd 3d XPS attenuation analysis was used to estimate the effective TiO_2_ shell thickness, giving a value of ∼1.0 nm (see details in the Supporting Information). The Ti 2p spectrum of Pd@TiO_2_/C shows a strong Ti^4+^ signal, with the 2p_3/2_ peak at 458.9 eV.^32^ The O 1s spectrum further confirms the presence of TiO_2_. While Pd/C shows a broad peak composed of oxygen in O=C bonds (531–532 eV), O–C and/or OH–C bonds (∼533 eV), and overlapping Pd 3p_3/2_ contributions (∼533–535 eV), Pd@TiO_2_/C displays a dominant lattice oxygen peak at ∼530.3 eV, characteristic of TiO_2_.^32,33^ The main carbon 1s peak at 284.5 eV can be assigned to the sp^2^ carbon. Additional features between 285.5 and 289 eV could arise from different kinds of carbon–oxygen bonding.^33^ These results confirm the formation of a core–shell Pd@TiO_2_ architecture and indicate that the TiO_2_ coating modulates the Pd electronic environment, stabilizing it in a reduced state.

### Electrochemical Characterization

In addition to the 24-cycle Pd@TiO_2_/C sample, 16- and 32-cycle samples were also synthesized to investigate the influence of the TiO_2_ shell thickness on HOR performance. The Pd and Ti K-edge XANES spectra of these samples closely overlap with those of the 24-cycle sample (Figure S6), indicating similar electronic structures. Furthermore, XPS analysis of the Pd 3d, Ti 2p, O 1s, and C 1s regions (Figure S7) shows spectral features comparable to those of the 24-cycle sample, confirming the preservation of the electronic environment across different TiO_2_ thicknesses.

The HOR performance of Pd/C and Pd@TiO_2_/C samples prepared with 16, 24, and 32 ALD cycles was evaluated by using a rotating disk electrode (RDE) system in alkaline media. Linear sweep voltammetry (LSV) was conducted in the negative scan direction at a slow scan rate of 5 mV s^–1^ to minimize contributions from hydrogen absorption. As shown in Figure 2a, LSV curves recorded in H_2_-saturated 0.1 M KOH at a rotation speed of 1600 rpm indicate that the 24-cycle Pd@TiO_2_/C sample delivers the highest anodic current density among the four catalysts. The LSVs for 24-cycle Pd@TiO_2_/C were repeated five times, and the average overpotential at 2 mA cm^–2^ is determined to be 65.2 ± 2.4 mV with a relative standard deviation of 3.7%, confirming the measurement repeatability (Figure S8). To eliminate mass transport effects, the limiting diffusion current densities (*j* ^d^) were evaluated.^34,35^ LSVs were measured at rotation speeds from 400 to 2400 rpm (Figure S9), and the corresponding Koutecky–Levich (K–L) plots at an overpotential of 0.35 V are shown in Figure S10. The extracted slopes for Pd/C, 16-cycle Pd@TiO_2_/C, 24-cycle Pd@TiO_2_/C, and 32-cycle Pd@TiO_2_/C samples were 14.60, 14.68, 14.50, and 14.65 cm^2^ mA^–1^ rpm^1/2^ respectively, corresponding to *j* ^d^ values of 2.74, 2.72, 2.76, and 2.73 mA cm^–2^ at 1600 rpm. Meanwhile, Pd mass fractions determined by inductively coupled plasma–optical emission spectroscopy (ICP-OES) were 4.45, 3.48, 3.46, and 3.45 wt % for Pd/C and the 16-, 24-, and 32-cycle Pd@TiO_2_/C samples, respectively. These values correspond to Pd loadings of 14.6, 11.4, 11.3, and 11.3 μg on the electrode. Based on K–L analysis, kinetic current densities (*j* ^k^) were calculated and normalized by Pd loading to obtain the mass-specific kinetic current densities (*j* ^k,m^).^36^ At an overpotential of 10 mV, the *j* ^k,m^ values were 12, 34, 45, and 21 mA mg_Pd_^–1^ for Pd/C, 16-, 24-, and 32-cycle Pd@TiO/C respectively.

**Figure 2:**
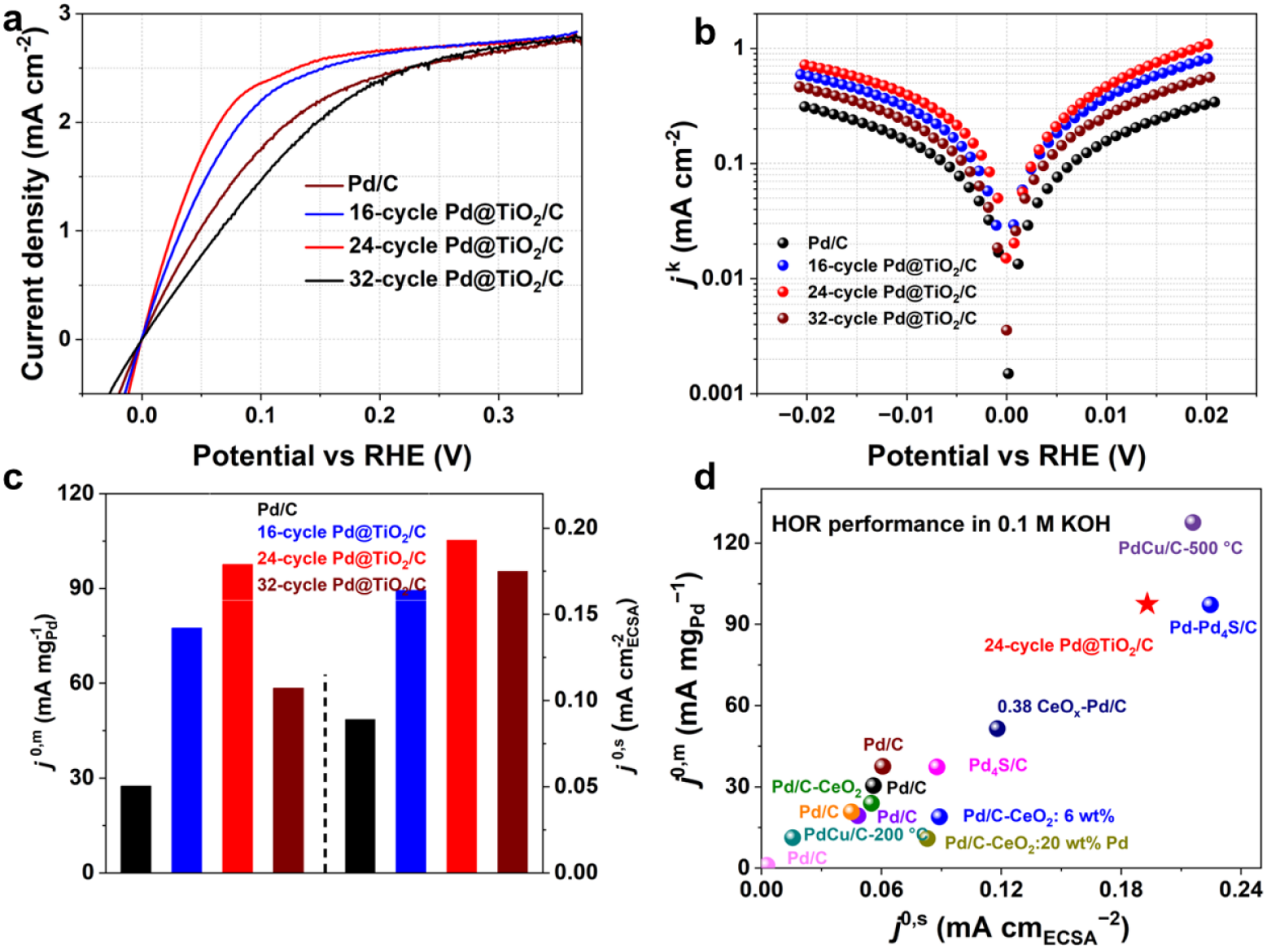
HOR performance and pathway analysis. **a)** The LSVs, **b)** Tafel plots, and **c)** mass activity and specific activity of Pd/C, 16-cycle Pd@TiO_2_/C, 24-cycle Pd@TiO_2_/C, and 32-cycle Pd@TiO_2_/C. **d)** Comparison of the HOR activity in 0.1 M KOH with Pd-based catalysts.

The exchange current density (*j* ^0^), a key indicator of intrinsic catalytic activity, was extracted via nonlinear fitting of Tafel plots of *j* ^k^ versus potential (Figure 2b and Figure S11).^37,38^ As shown in Figure 2c and Table S2, the 24-cycle Pd@TiO_2_/C exhibits the highest mass-specific exchange current density (*j* ^0,m^) of 98 mA mg_Pd_^–1^, which is over three times higher than that of Pd/C (28 mA mg_Pd_^–1^), and surpasses that of the 16-cycle Pd@TiO_2_/C (78 mA mg_Pd_^–1^) and 32-cycle Pd@TiO_2_/C (59 mA mg_Pd_^–1^) samples. Additionally, CO stripping measurements (Figure S12) were employed to estimate the electrochemically active surface area (ECSA).^39,40^ After normalization by ECSA, the specific exchange current densities (*j* ^0,s^) were calculated. The 24-cycle Pd@TiO_2_/C shows the highest value of 0.193 mA cm_ECSA_^–2^, compared to Pd/C (0.089 mA cm_ECSA_^–2^), 16-cycle Pd@TiO_2_/C (0.164 mA cm_ECSA_^–2^), and 32-cycle Pd@TiO_2_/C (0.175 mA cm_ECSA_^–2^). The HOR performance of 24-cycle Pd@TiO_2_/C is also higher than most of the reported Pd-based HOR electrocatalysts in Figure 2d and Table S3.^39,41–44^ These results collectively demonstrate that the TiO_2_ shell significantly enhances the HOR activity of the Pd/C catalysts.

### Degradation Mechanism Study

Identical location transmission electron microscopy (IL-TEM) was employed to gain a deeper understanding of the degradation pathway. As shown in Figure 3a, a custom-designed polyether ether ketone clamp was used to secure the TEM grid onto a glassy carbon RDE electrode, ensuring both mechanical stability and electrical contact while leaving the central region exposed. This configuration enabled electrochemical durability testing while maintaining the precise positioning of the grid for repeated imaging.

**Figure 3:**
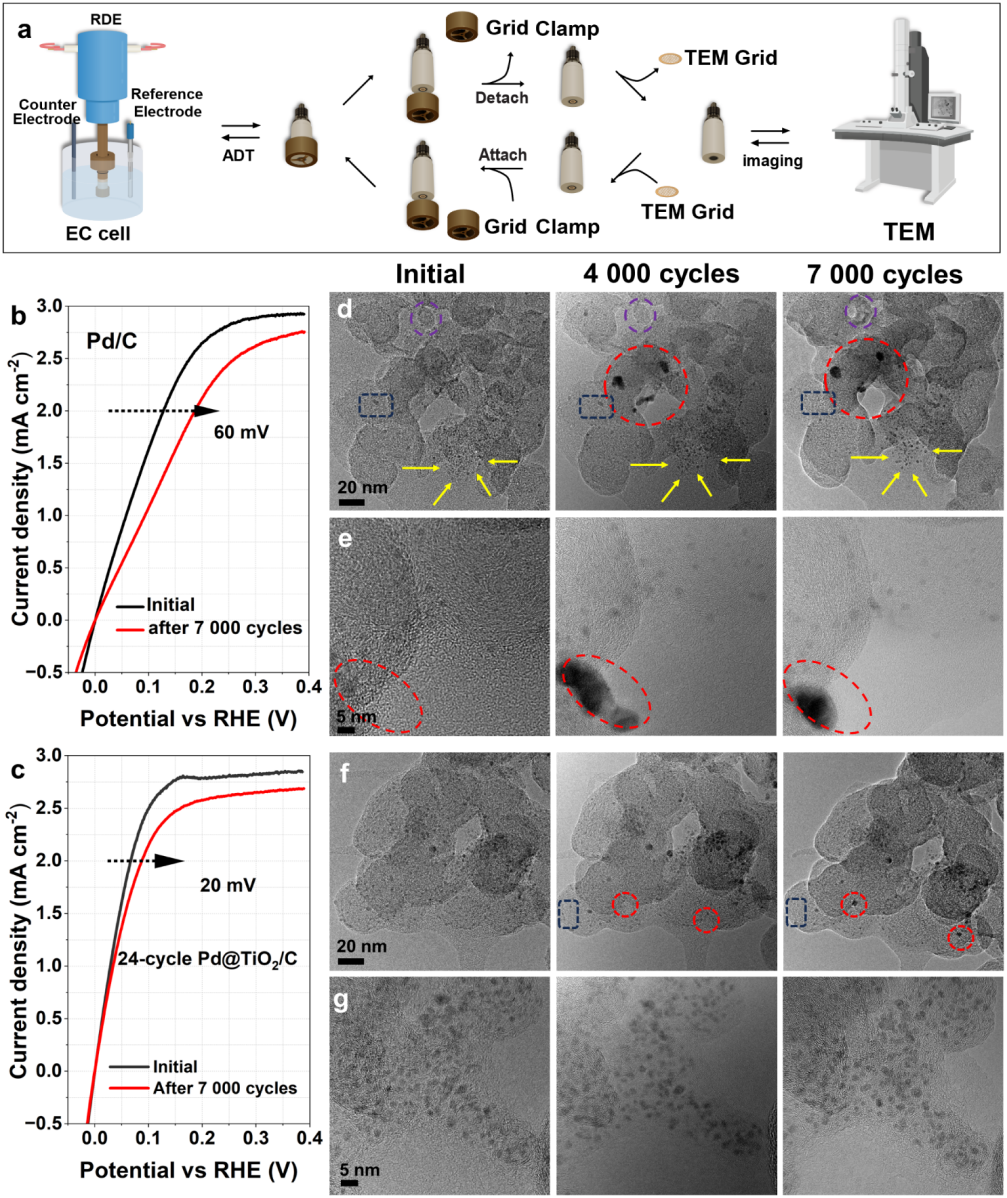
Degradation mechanism study for Pd/C and 24-cycle Pd@TiO_2_/C. **a)** Schematic of the IL-TEM experiment. LSV curves of **b)** Pd/C and **c)** 24-cycle Pd@TiO_2_/C before and after 7 000 ADT cycles in H_2_-saturated 0.1 M KOH. **d)** and **e)** IL-TEM images of two regions from the Pd/C sample at the initial state (left), after 4,000 cycles (middle), and after 7,000 cycles (right). **f)** and **g)** IL-TEM images of two regions from the Pd@TiO_2_/C sample at the initial, 4 000, and 7 000 cycles (left to right).

Electrochemical stability was evaluated by accelerated durability tests (ADT) conducted in H_2_-saturated 0.1 M KOH using cyclic voltammetry at a scan rate of 100 mV s^–1^ without the use of the TEM grid. After 7 000 potential cycles, HOR performance was reassessed using LSV in H_2_-saturated alkaline solution (Figure S13). As shown in Figure 3b and c, the Pd/C catalyst exhibits a 60 mV increase in overpotential at a current density of 2 mA cm^–2^, while the 24-cycle Pd@TiO_2_/C shows only a 20 mV increase, demonstrating markedly improved durability after the TiO_2_ coating. IL-TEM characterization was then performed on 5 wt % Pd/C and the corresponding 24-cycle Pd@TiO_2_/C. Although nanoparticle detachment was clearly observed in the 5 wt % samples after ADT (Figure S14), the low nanoparticle density may introduce substantial variability in particle selection and tracking. To enable more reliable analysis, Pd/C and Pd@TiO_2_/C with a higher Pd loading of 15 wt % were prepared and examined.

IL-TEM images in Figure 3d and e present two representative regions of the Pd/C at distinct magnifications, imaged at the initial state, after 4 000 cycles, and after 7 000 cycles (left to right in each panel). These images reveal pronounced morphological transformations of the Pd particles, while the carbon support mostly retains its structure. The red dashed circles highlight the emergence of large Pd particles after 4 000 cycles, indicating aggregation through coalescence. Some oversized particles disappear after 7 000 cycles, suggesting detachment from the carbon support once the particle size exceeds the anchoring capacity of the substrate. Particle growth is attributed to a combination of Ostwald ripening and the merging of adjacent nanoparticles, as evidenced by the dark blue dashed circles and yellow arrow-marked regions, respectively. The dark blue circles highlight the progressive disappearance of particles, indicating the electrochemical dissolution of Pd during cycling. The yellow arrows indicate regions where particle boundaries become increasingly well-defined, consistent with coalescence-driven growth through particle fusion. Additionally, morphological changes in the carbon support are observed within the purple circle at 7 000 cycles. However, as no systematic carbon degradation is detected across multiple regions, this feature is most likely due to localized mechanical stress, causing partial carbon detachment during operation.

In contrast, the 24-cycle Pd@TiO_2_/C exhibits markedly improved structural stability. Figure 3f and g show IL-TEM images of distinct regions at the initial state, after 4 000 cycles, and after 7 000 cycles (left to right). In Figure 3f, only minor variations are observed: new particles appear in the red-circled region, and a few disappear from the blue-dashed areas after 7 000 cycles. In Figure 3g, nanoparticle distribution and morphology remain virtually unchanged throughout the test. These localized changes likely reflect surface reconstruction or minor migration of Pd@TiO_2_ nanoparticles but are minimal compared to the extensive degradation observed in Pd/C. Quantitative particle size analysis (Figure S15) further supports these observations. For Pd/C, the average particle size decreases from 2.3 ± 0.3 nm to 1.7 ± 0.3 nm after 7 000 cycles, while Pd@TiO_2_/C exhibits only a slight reduction, from 2.5 ± 0.3 nm to 2.2 ± 0.3 nm, suggesting better structural preservation. These statistics are based on measurements of more than 60 nanoparticles from high-resolution TEM images, excluding newly formed oversized particles. The slight decrease in average size aligns with the emergence of large particles observed in Figure 3, suggesting that localized coalescence and dissolution contribute to nanoparticle degradation. More IL-TEM images for Pd/C and Pd@TiO_2_/C are provided in Figures S16–S21.

Overall, a growth–detachment degradation mechanism is proposed, supported by both direct imaging and statistical analysis. The TiO_2_ shell provides physical confinement, effectively suppressing excessive particle migration and coalescence. This structural stabilization reduces the likelihood of forming oversized nanoparticles and thus minimizes detachment from the support. In addition, the TiO_2_ shell acts as a protective barrier against the electrochemical dissolution of Pd, slowing size redistribution processes that are common in uncoated catalysts.

### Reaction Mechanism Study

Operando XAS was performed to further elucidate the reaction mechanism and the respective roles of the Pd core and TiO_2_ shell in the HOR under AEMFC-relevant conditions. A custom-designed operando cell was built to enable simultaneous electrochemical operation and XAS measurements. As illustrated in Figure 4a, the cell has a stacked architecture with a PTFE gasket and an integrated heater containing a rectangular aperture for X-ray transmission. Carbon-based gas diffusion layers (GDLs) and glassy carbon flow plates were used, as both are transparent to X-rays at the Pd K-edge (∼24.35 keV) and Ti K-edge (∼4.97 keV). For safety and consistency, measurements were carried out at room temperature and 40 °C under a continuous H_2_ and O_2_ flow (10 mL min^–1^ each). The XAS data were collected 3–5 min after initiating the electrochemical procedure to ensure quasi-steady-state conditions at each measurement point. The cell configuration is shown in Figure 4b, with the anode facing the incident X-ray beam to optimize fluorescence detection.

**Figure 4:**
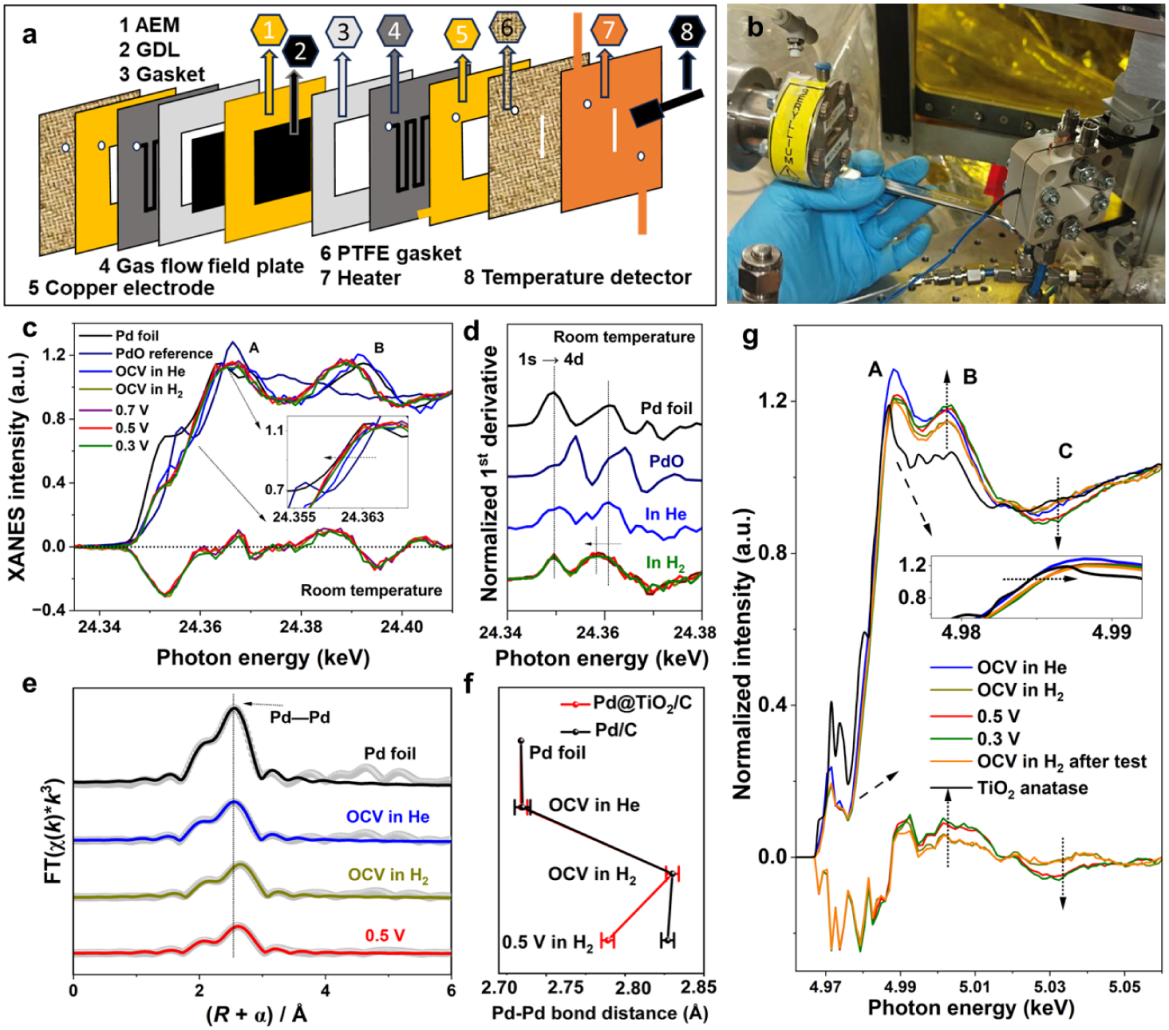
Operando XAS measurement for Pd@TiO_2_/C. **a)** Schematic of the custom-designed operando XAS cell. **b)** Photograph of the operando cell mounted on the beamline stage. **c)** Normalized Pd K-edge XANES spectra of Pd@TiO_2_/C under OCV in He and H_2_ and at applied voltages of 0.7 V, 0.5 V, and 0.3 V in H_2_ at room temperature; difference spectra shown below. **d)** First derivative of the XANES spectra in **c)**. **e)** Fourier-transformed EXAFS spectra and corresponding fits for the Pd foil and Pd@TiO_2_/C under the OCV in He, the OCV in H_2_, and 0.5 V in H_2_. **f)** The comparison of Pd–Pd bond distance for Pd/C and Pd@TiO_2_/C at OCV in He, OCV in H_2_, and 0.5 V in H_2_. The data for Pd/C at OCV in H_2_ are obtained from reported literature.^45,47^ **g)** Ti K-edge XANES spectra of the TiO_2_ anatase reference, Pd@TiO_2_/C under OCV in He, OCV in H_2_, and working voltages (0.5 V and 0.3 V in H_2_).

The 24-cycle Pd@TiO_2_/C was first examined by Pd K-edge XANES under a range of electrochemical conditions. A Pd foil and PdO served as reference materials representing metallic and oxidized Pd states, respectively. The operando setup was validated by the overlap between the XANES spectra of Pd@TiO_2_/C at OCV in He (both sides) and its ex situ counterpart (Figure S22). Subsequent measurements were acquired under OCV in H_2_/O_2_, followed by applied voltages of 0.7 V, 0.5 V, and 0.3 V in H_2_/O_2_. For the sake of simplicity, the following discussion refers only to the gas supplied to the anode. As shown in Figure 4c, the peak A at 24.37 keV and the peak B at 24.39 keV shift to lower energies under H_2_ compared to He, and the peak B exhibits a relative decrease in intensity. These changes are attributed to the hydrogen diffusion into the Pd lattice and the resulting increase in the Pd–Pd bond distance, indicating the formation of Pd–H_*x*_ species.^32,45^ Only minor changes are observed under applied potentials, suggesting that Pd remains in a hydrogen-absorbed metallic state under operational conditions (see the difference spectra at the bottom of Figure 4c). First-derivative spectra in Figure 4d further support this interpretation. The first peak in the derivative spectra near 24.35 keV is attributed to the 1s → 4d electronic transition, which serves as an indicator of the valence state of Pd. The peak positions under OCV in H_2_ and all applied voltages align with that of metallic Pd, indicating a stable Pd valence state during HOR. The second peak at 24.36 keV shifts to lower energy and broadens when switching to H_2_, associated with the mixing of Pd d-states with hydrogen s- and p-unoccupied states.^46^

In addition, operando Pd K-edge XANES was repeated at 40 °C to confirm reproducibility under more representative conditions. As shown in Figure S23, the spectral evolution under OCV in H_2_ and at applied voltages of 0.7, 0.5, and 0.3 V in H_2_ closely mirrors the room-temperature behavior, with even stronger spectral overlap. For comparison, operando XANES was also conducted on Pd/C under identical conditions. The Pd/C exhibits similar shifts in the edge position and white line intensity (Figure S24), consistent with hydrogen absorption. The ex situ XANES of Pd@TiO_2_/C and Pd/C before and after the operando measurements (Figure S25) show negligible spectral differences, indicating that no beam-induced damage occurred during data collection.

Fourier-transformed extended X-ray absorption fine structure (EXAFS) spectra were collected to probe the local coordination environment of Pd@TiO_2_/C under OCV in He, OCV in H_2_ and at 0.5 V in H_2_ (Figure 4e). All spectra exhibit a dominant peak at ∼2.6–2.9 Å, corresponding to first-shell Pd–Pd coordination. Quantitative fitting was performed for the first Pd–Pd shell, assuming single scattering. A distinct Pd–H scattering path could not be resolved due to the weak backscattering amplitude of hydrogen and the limited signal-to-noise ratio. The coordination numbers (*N*) are 8.2, 8.5, and 8.3 for Pd@TiO_2_/C under OCV in He, OCV in H_2_, and at 0.5 V in H_2_, respectively, which are consistent with the sample in ex-situ condition (Table S1). The Pd–Pd bond distance provides a sensitive indicator for PdH_*x*_ formation and decomposition.^47^ Under the OCV in He, the bond distance is 2.734 Å, closely matching that in the Pd foil (Figure 4f). Upon H_2_ exposure, it expands to 2.830 Å, reflecting the lattice expansion caused by hydrogen absorption and the formation of a PdH_*x*_ phase. The observed bond distance aligns with literature-reported values for Pd under comparable conditions, indicating that the TiO_2_ shell does not suppress the lattice expansion.^47,48^ When a potential of 0.5 V is applied, the Pd–Pd distance for Pd@TiO_2_/C decreases to 2.789 Å, whereas Pd/C attains 2.827 Å, significantly larger under the same conditions (Figure S26 and Table S1). The HOR and hydrogen absorption compete on core–shell Pd@TiO_2_/C: HOR depletes lattice hydrogen via spillover, whereas absorption increases the PdH_*x*_ content. The shorter Pd–Pd distance observed for Pd@TiO_2_/C at 0.5 V in H_2_ indicates that the TiO_2_ shell promotes HOR by facilitating hydrogen desorption. The bond distance change is also reflected in the oscillation pattern in *k*-space (Figure S27).

To probe the chemical state and functional role of the TiO_2_ shell, operando Ti K-edge XANES was performed on Pd@TiO_2_/C under OCV in He and H_2_, followed by applied voltages of 0.5 V and 0.3 V in H_2_. After voltage application, the cell was returned to the OCV in H_2_ to verify spectral reversibility. Ex situ anatase TiO_2_ was used as a reference. As shown in Figure 4g, all spectra display three characteristic features (peaks A–C) typical of anatase TiO_2_. Under the OCV in He, the absorption edge and peak positions align well with the Ti^4+^ reference. Upon H_2_ exposure, the Ti K absorption edge shifts to a higher energy, indicating a more oxidized local environment around Ti. This shift is likely due to hydrogen adsorption on Pd, which lowers the Fermi level of the Pd core.^49,50^ To equilibrate the Fermi levels at the PdH_*x*_–TiO_2_ interface, electrons transfer from the TiO_2_ shell into the hydrogenated Pd phase.^51,52^ This electron depletion near the TiO_2_ surface suppresses oxygen vacancy states, shifting the Ti centers toward a more Ti^4+^-like configuration, reflected in the Ti K-edge XANES as a subtle positive shift in the absorption edge.^53–55^ The structural change of TiO_2_ is supported by the diminished pre-edge feature under OCV in H_2_, indicating a lower concentration of oxygen vacancies and structural defects.^48^ Under applied potentials of 0.5 V and 0.3 V in H_2_, the pre-edge and rising-edge features remain unchanged compared to those of the OCV in H_2_, indicating that the Ti oxidation state is maintained. However, the peak B intensity increases, while the peak C decreases, suggesting alterations in the local coordination environment. The peaks B and C correspond to the first two postedge multiple-scattering resonances of the TiO_6_ octahedra, involving Ti–O–Ti pathways and medium-range structural order.^27,56^ Under AEMFC working potentials, the accelerated HOR rate leads to increased local H_2_O generation and a higher flux of OH^–^, resulting in more extensive hydroxylation of the TiO_2_ shell (formation of Ti–OH/H_2_O adlayers). This hydroxylation perturbs the Ti–O–Ti network by altering bond angles and second-shell Ti coordination, directly impacting the postedge multiple-scattering features. In the Ti K-edge XANES, these modifications manifest as a redistribution of intensity for the peaks B and C, indicating that the TiO_2_ shell enhances reaction kinetics by stabilizing OH^–^ intermediates at the metal–oxide interface.^57,58^

In summary, the HOR on Pd@TiO_2_/C follows a dual-site mechanism at the surface-accessible Pd–TiO_2_ interfacial boundary in which the Pd core and TiO_2_ shell play complementary roles. The Pd core functions as the hydrogen adsorption and absorption site, where H_2_ molecules dissociate and form hydride species. In parallel, the TiO_2_ shell not only promotes hydrogen desorption from Pd but also serves as the OH^–^ adsorption site, stabilizing hydroxyl intermediates that are essential for the Heyrovsky and Volmer steps in alkaline media. In addition, electron interactions between the hydrogen-absorbing Pd core and the TiO_2_ shell promote charge redistribution and stabilize the interface. Together, this cooperative mechanism accelerates the overall HOR kinetics, thereby overcoming the intrinsic limitations of Pd alone in alkaline environments. Validating the enhanced kinetics, Pd@TiO_2_/C exhibits improved AEMFC performance relative to that of Pd/C (Figure S28).

### Reaction Pathway Analysis

Electrochemical impedance spectroscopy (EIS) was used to elucidate kinetics by evaluating the relative contributions of the Heyrovsky–Volmer and Tafel–Volmer pathways to the HOR. Nyquist plots and corresponding fitted equivalent circuits for the 24-cycle Pd@TiO_2_/C and pure Pd/C samples are presented in Figure 5a and Figure S29, respectively. The equivalent circuit model in Figure 5a comprises subcircuits representing the Heyrovsky–Volmer and Tafel–Volmer pathways, and it effectively captures the impedance response over the investigated potential range.^59^ In this model, *R*_u_ denotes the electrolyte resistance; *R*_ct1_ is the charge transfer resistance for the Tafel–Volmer pathway; *R*_s_ and *C*_s_ represent adsorption resistance and capacitance, respectively; *R*_ct2_ corresponds to the charge transfer resistance for the Heyrovsky–Volmer pathway; and *Z*_Diff_ represents the Warburg diffusion impedance. The ratio *R*_ct2_/*R*_ct1_ indicates the relative contributions of the Heyrovsky–Volmer and Tafel–Volmer mechanisms to the overall current without significant diffusion limitations.^59^ These ratios are plotted in Figure 5b as a function of the electrode potential for the 24-cycle Pd@TiO_2_/C and pure Pd/C. Neither mechanism dominates across the investigated potential range, indicating complex mixed kinetics. The *R*_ct2_/*R*_ct1_ ratios for the 24-cycle Pd@TiO_2_/C at 0, 10, 20, 30, 40, and 50 mV_RHE_ are 1.22, 1.12, 0.75, 1.01, 0.77, and 0.98, respectively, which are consistently lower than those of pure Pd/C at the same potentials. This suggests that TiO_2_ facilitates the Heyrovsky step, leading to a greater contribution from the Heyrovsky–Volmer pathway after TiO_2_ deposition. These findings align with operando XAS measurements, which indicate that the TiO_2_ shell accelerates the Volmer and Heyrovsky steps, thereby enhancing the overall reaction kinetics.

**Figure 5:**
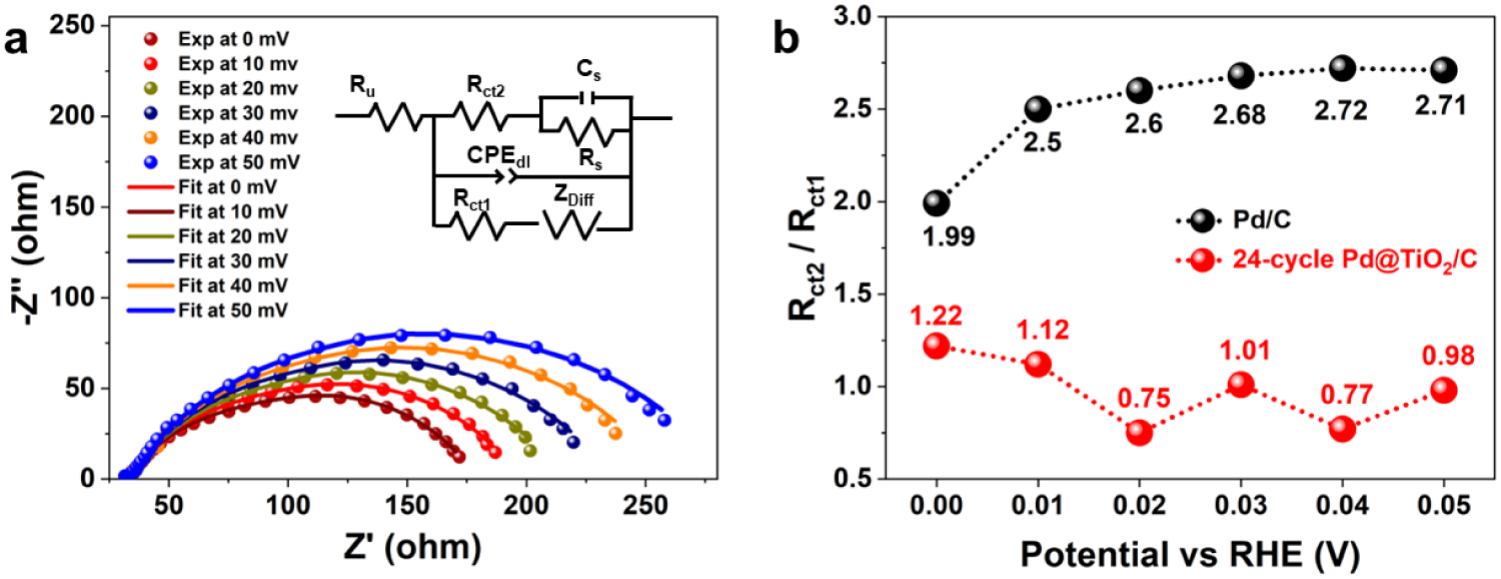
a) EIS data at different potentials and corresponding fitting results for 24-cycle Pd@TiO_2_/C. **b)** Ratio *R*_ct2_/*R*_ct1_ for Pd/C and 24-cycle Pd@TiO_2_/C.

### Understanding CO Reaction on Pd@TiO_2_/C

CO is a well-known poison for Pd-based anodes in AEMFCs; even parts per million level CO in H_2_ streams can perturb surface adsorption equilibria. To assess the influence of CO on the Pd@TiO_2_/C interface, operando XAS was performed on Pd@TiO_2_/C under controlled CO exposure. Pd K-edge XANES was conducted under a sequential gas exposure protocol: first, an OCV in He, then an OCV in H_2_, followed by 0.5 V in a mixed H_2_/CO atmosphere (10 mL min^–1^ H_2_ and 0.02 mL min^–1^ CO), and finally 0.5 V in pure CO (2 mL min^–1^). As shown in Figure 6a, the XANES spectra under mixed and pure CO closely resemble those obtained under OCV in H_2_. This suggests that CO adsorption does not markedly alter the electronic structure of Pd and that the Pd core remains in a reduced state. The persistence of postcrest features (e.g., the peaks B and C) indicates that lattice hydrogen is retained, and its formation is not prevented by CO adsorption. The corresponding first-derivative spectra (Figure 6b) provide clearer evidence of the CO surface adsorption. A distinct shift (∼1 eV) to lower energy of the first peak is observed for mixed and pure CO relative to the Pd foil and OCV in H_2_, indicating that CO adsorbs on the Pd surface. This behavior is consistent with observations in the Pd/C samples under CO exposure (Figure S30), where a similar derivative shift validates this feature as a fingerprint for CO adsorption.

**Figure 6:**
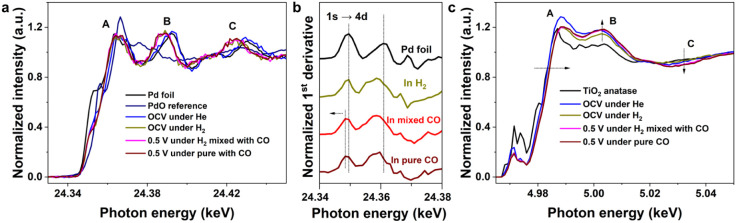
Operando XAS characterization of Pd@TiO_2_/C under CO exposure. **a)** Normalized Pd K-edge XANES spectra measured under OCV in He, OCV in H_2_, 0.5 V in mixed H_2_/CO (10 mL/min H_2_, 0.02 mL/min CO), and 0.5 V in pure CO (2 mL/min), the Pd foil, and PdO references. **b)** First derivative of the XANES spectra in **a)**. **c)** Ti K-edge XANES spectra under the same gas conditions, compared with the TiO_2_ anatase reference.

Figure 6c shows Ti K-edge XANES spectra collected under the same conditions. The rising edge under 0.5 V in mixed or pure CO overlaps with that observed under the OCV in H_2_ and remains shifted to higher energy compared to the OCV in He. This originates from the lattice H remaining when CO adsorbs on the Pd surface, leading to electron transfer from the TiO_2_ shell to the Pd core. Furthermore, the intensity of peak B increases, while that of peak C decreases upon CO exposure, suggesting that TiO_2_ remains in the OH^–^ adsorbed state and this process is not affected by CO adsorption. These results collectively demonstrate that although CO adsorbs on the Pd surface, it does not displace lattice hydrogen in the Pd core or impair the functional role of the TiO_2_ shell.

## Conclusion

In conclusion, we developed a core–shell Pd@TiO_2_/C catalyst for HOR and systematically investigated its degradation pathways and reaction mechanisms under the operating conditions. The catalyst demonstrates markedly enhanced stability and activity for the alkaline HOR compared with uncoated Pd/C owing to both the protective and functional roles of the TiO_2_ shell. IL-TEM reveals a growth–detachment degradation pathway during ADT, in which Pd/C undergoes severe nanoparticle agglomeration and dissolution, whereas Pd@TiO_2_/C largely preserves its structural integrity. Operando XAS under realistic AEMFC conditions demonstrates the dynamic evolution of the PdH_*x*_ phase. The formation of PdH_*x*_ lowers the Fermi level of Pd, driving electron transfer from the TiO_2_ shell to the Pd core and shifting Ti toward a slightly higher oxidation state than Ti^4+^. The TiO_2_ shell promotes hydrogen desorption while suppressing PdH_*x*_ formation, thereby enhancing reaction kinetics, while simultaneously providing OH^–^ adsorption sites that stabilize hydroxyl intermediates essential for alkaline HOR. These findings establish the dual function of TiO_2_ as both a structural stabilizer and an active interfacial component, providing mechanistic insights and design principles for developing durable, high-performance HOR catalysts.

## Supplementary Material



## Notes

Additional experimental methods (characterization, fuel cell testing, IL-TEM, operando XAS), supplementary figures (TEM, XRD, XPS, XAS, HOR performance), and tables of EXAFS fitting parameters and HOR benchmarking data (PDF)

## #

Sunwoda Mobility Energy Technology Co., Ltd., Shenzhen, Guangdong, 518000, China
