# Supplementary material for: Decoupling H‑Release and OH– Management at Pd@TiO2 Interfaces for Efficient Alkaline Hydrogen Oxidation Reaction
Source: ACS Catal. 2026 Feb 12;16(4):3699–710. doi: 10.1021/acscatal.5c08285 (PMC12930519; doi:10.1021/acscatal.5c08285)
Supplement: Supplementary file 1 [file cs5c08285_si_001.pdf]

Supporting Information

Decoupling H-Release and OH^{-} Management at Pd@TiO_2 Interfaces for efficient Alkaline Hydrogen Oxidation Reaction

*Benjin Jin [a], Antti-Jussi Kallio [b], Nils Rieger [c], Vasyl Marchuk [d], Cedric Schiwek [a], Junjie Shi [a†], Jani Sainio [e], Hua Jiang [e], Amine Hammouali [a], Jefina A S Koivuniemi [b], Nana Han [a], Björn Wickman [c], Simo Huotari [b], and Tanja Kallio [a *]*

[a] *Department of Chemistry and Materials Science, School of Chemical Engineering, Aalto University, P.O. Box 16100, FI-00076 Aalto, Finland.*

[b] *Department of Physics, University of Helsinki, P. O. Box 64, FI-00014, Finland*

[c] *Department of Physics and Competence Centre for Catalysis, Chalmers University of Technology, Göteborg SE-412 96, Sweden*

[d] *ESRF, The European Synchrotron, Grenoble 38043, France*

[e] *Department of Applied Physics, School of Science, Aalto University, P.O. Box 15100, FI-00076 Aalto, Finland*

[*] E-mail: tanja.kallio@aalto.fi

[†] *Sunwoda Mobility Energy Technology Co., Ltd, Shenzhen Guangdong, 518000, China.*

Table of Contents

1. Characterizations 3

X-ray photoelectron spectroscopy (XPS) 3

Inductively Coupled Plasma Optical Emission Spectrometry (ICP-OES) 3

2. Fuel cell measurements 4

3. Identical location transmission electron microscopy (IL-TEM) 4

4. Operando X-ray absorption spectroscopy 5

Cell structure 5

Electrochemical operation 6

Operando XAS measurements 6

5. Supplementary Figures 8

6. Supplementary Tables 38

7. Reference 41

1. Characterizations

X-ray photoelectron spectroscopy (XPS)

The sp^2 C 1s peak at 284.4 eV was used as a binding energy reference. All C 1s spectra appeared to be aligned to this position and thus no additional binding energy correction was applied. The elemental composition was determined from peak areas of high-resolution core level spectra after Shirley background subtraction using equipment specific sensitivity factors. Because of the severe overlap between the O 1s and Pd $3p_{3/2}$ peaks the O 2s peak was used in the determination of the elemental composition.

XPS attenuation analysis: the XPS spectra were acquired in normal emission ($\cos\theta = 1$, with the emission angle defined relative to the surface normal). In this geometry, the Pd 3d intensity decays exponentially with the TiO_2 overlayer thickness d according to the standard overlayer model:

$$I_{Pd} = I_{Pd}^{0} \exp\left(-\frac{d}{\lambda_{Pd\ in\ TiO2}}\right)$$

$$d = \lambda_{Pd\ in\ TiO2} \ln\frac{I_{Pd}^{0}}{I_{Pd}}$$

where I_{Pd}^{0} is the Pd 3d doublet area for bare Pd/C and I_{Pd} is that after TiO_2 ALD. $\lambda_{Pd\ in\ TiO2}$ is the effective attenuation length for Pd 3d photoelectrons traversing TiO_2. Based on literature reported values in TiO_2, we use $\lambda_{Pd\ in\ TiO2} = 1.6\ nm$.[1]

The Pd 3d doublet areas were obtained by integrating the background-corrected spectra (334–345 eV) with the Origin software. Using the integrated Pd 3d doublet areas (bare Pd/C: 16457 a.u.; 24 cycles: 8776 a.u.), we obtain the effective thickness of the TiO_2 shell and also the growth per cycle (GPC) are:

24 cycles: $d_{24} \approx 1.01\ nm$ $\qquad$ $GPC \approx \frac{1.01}{24} \approx 0.042\ nm\ cycle^{-1}$

Inductively Coupled Plasma Optical Emission Spectrometry (ICP-OES)

The samples were digested in an aqueous solution of HCl: HNO_3 (3:1 volume ratio) by heating the mixture in a microwave oven (Speedwave XPERT). The solution was introduced into a microwave oven inside a closed vessel and heated up to 210 °C for 20 min and then held at

210 °C for 30 min. Subsequently, the digestion solution was diluted to 50 mL with MQ water. The 50 mL solution was further diluted 5 times before the ICP-OES measurements. The digested sample weighed around 10 mg. Each sample was measured twice or thrice. ICP-OES detects characteristic wavelengths of each element after their energy transition by the plasma. The wavelengths used for Pd are 229.651, 340.458, and 351.694 nm.

2. Fuel cell measurements

FAA-3-50 was used as an anion exchange membrane. The membrane was first soaked in 0.5 M NaCl solution for 24 h to remove any residual additives, followed by immersion in 0.5 M KOH for an additional 24 h to exchange the fluoride ions with hydroxide (OH^-) ions. After ion exchange, the membrane was stored in deionized water. Prior to use, the water was purged with N_2 for 30 minutes to remove dissolved CO_2. Sigracet 22 BB was employed as the gas diffusion layer (GDL). The catalyst layer was applied onto the GDL surface by airbrushing. Typically, the catalyst powder was mixed with 5 wt% FAA-3 ionomer solution, with the ionomer content adjusted to 30 wt% relative to the total catalyst ink. The catalyst loading was controlled at 0.4 mg_{Pd} cm^{-2} for the anode and 0.5 mg_{Pt} cm^{-2} for the cathode. After deposition, the GDL and membrane were pressed together under a pressure of 2 tons for 20 s at room temperature to form the membrane electrode assembly (MEA). The MEA was operated under 100% humidified conditions. Hydrogen was supplied to the anode at a flow rate of 100 sccm, and oxygen was fed to the cathode at 50 sccm.

3. Identical location transmission electron microscopy (IL-TEM)

Catalyst ink was prepared following the same protocol as used for covering the GC disk samples. Specifically, 970 µL of isopropanol and 30 µL of a Nafion solution (Nafion® 117, 5 wt. % solution, Aldrich) were mixed with 4 mg of catalyst powder. From this ink, 0.5 µL was drop-casted onto TEM grids (UltrAuFoil, Quantifoil) and subsequently dried under ambient conditions. Initial high-resolution TEM (HRTEM) imaging was performed using a FEI Titan microscope operated at 300 kV. Following imaging, each grid was individually mounted onto a RDE head equipped with a blank GC disk. A custom-designed cap made from polyether ether ketone (PEEK) was used to clamp the grid onto the GC disk. This design ensured that the central region of the grid remained fully exposed, while fixation occurred at the grid's rim. Contact between the clamp and the grid was achieved via small pins that applied gentle pressure

to secure the grid without obstructing the central area. This way, the grid was pressed against the GC electrode which ensured electrical contact to the grid. With the grid mounted on the RDE head, ADT was conducted in the same fashion as for the GC disks covered with catalyst ink. The RDE setup (Pine Research) was connected to a potentiostat (SP-300, BioLogic), and the potential was cycled between 0.0 V and 0.4 V vs. the reversible hydrogen electrode (RHE) at a scan rate of $100\,mV\,s^{-1}$. The ADT was performed in a Teflon electrochemical cell containing 0.1 M KOH (Merck KGaA), using a SPK-grade graphite rod (6.15 mm diameter, Thermo Scientific Chemicals) as the counter electrode and a Hg/HgO reference electrode (CHI152, CH Instruments, Inc.). For each experiment, the reference electrode's potential relative to the RHE was confirmed using a platinum wire immersed in a H_2-saturated electrolyte solution and measuring the potential at zero current. Prior to testing, the electrolyte was purged with high-purity argon (grade N6.0, Strandmøllen) for at least 20 minutes. During the ADT, argon bubbling was halted to prevent bubble formation on the electrode, but the cell was kept under argon atmosphere to avoid oxygen exposure. After 4 000 and 7 000 potential cycles, respectively, the ADT was interrupted, and the grid was retrieved for intermediate and final identical location TEM (IL-TEM) imaging. Following imaging after 4 000 cycles, the grid was reinserted into the RDE setup, and the ADT was resumed.

4. Operando X-ray absorption spectroscopy

Cell structure

This cell builds upon and improves the design presented in our earlier work. The core of the cell consists of a MEA with a 15 × 15 mm² active area, comprising an anion exchange membrane sandwiched between catalyst-coated electrodes and gas diffusion layers. The MEA is enclosed between laser-micro-machined glassy carbon flow channels, which provide both gas delivery and X-ray transparency at Pd and Ti K-edge energies. The outer casing is machined from chemically resistant PEEK, with interchangeable configurations tailored for either transmission or fluorescence XAS modes. One shell design includes 55° beveled edges and a rear groove to accommodate a silicon drift detector close to the sample for enhanced fluorescence signal collection. Polytetrafluoroethylene gaskets are used to seal the MEA and isolate the gas compartments, while copper foil back contacts provide electrical connection. A thin-film Kapton heater is integrated into the stack for temperature control, and a platinum resistance temperature detector is placed in contact with the stack for real-time monitoring. This modular, gas-tight design enables precise control over electrochemical environment,

temperature, and X-ray beam access, making it ideal for operando XAS measurements of catalyst structure and electronic state under realistic AEMFC operation.

Electrochemical operation

The catalyst ink for the anode was prepared by dispersing Pd-based catalyst powder in a mixture of deionized water and isopropyl alcohol, with 30 wt% anion exchange ionomer loading. The suspension was stirred overnight and then ultrasonicated for 30 minutes to ensure a homogeneous slurry. The ink was air-sprayed onto carbon paper, yielding a Pd loading of 1 mg cm^{-2}. For the cathode, commercial Pt/C (1 mg Pt cm^{-2}) was used. The anode and cathode electrodes were pressed onto either side of an alkaline exchange membrane (Fumasep FAA-3-50 or PiperION^{TM} A80-HCO_3) at room temperature under 2 MPa pressure for 20 s, forming a MEA with a geometric area of 2.25 cm^2. Electrochemical measurements were carried out using a Bio-Logic SP240 potentiostat at both room temperature and 40 °C, with temperature regulated using a Kapton-based thin-film heater and monitored using a platinum resistance temperature detector in contact with the cell stack. Hydrogen (10 mL min^{-1}) and oxygen (10 mL min^{-1}) were supplied to the anode and cathode, respectively. All gases were humidified to 100% relative humidity prior to entering the cell. The gas flows were controlled via precision mass flow controllers. Prior to XAS measurements, open-circuit voltage (OCV) and linear sweep voltammetry (LSV, 10 mV s^{-1}) were recorded to ensure proper MEA operation. For XAS data collection, the system was first measured with He gas (10 mL min^{-1} on both electrodes) under OCV conditions. This was followed by measurements under H_2/O_2 operation at OCV, and then under applied voltages of 0.7 V, 0.5 V, and 0.3 V under H_2/O_2. To evaluate CO effects, two anode gas feeds were used: a mixed stream of H_2 (10 mL min^{-1}) and CO (0.02 mL min^{-1}), and pure CO (2 mL min^{-1}). XAS spectra were collected at steady-state for each condition.

Operando XAS measurements

Operando X-ray absorption spectroscopy (XAS) measurements were carried out at beamline ID26 of the European Synchrotron Radiation Facility (ESRF). Synchrotron light was generated using three consecutive 1.6 m undulators. Measurements at the Ti K-edge were performed at the fundamental undulator harmonic. Higher harmonics were rejected by Si mirrors with 2.5 mrad incidence angles. For the measurements on the Pd K-edge, the fifth undulator harmonic was selected by filtering out lower energies by a set of diamond attenuators. At their energies, Pt coating of the mirrors was utilized with 2.5 mrad incidence angles. Si(111) monochromator

was used for measurements at the Ti K edge, and a Si(311) monochromator provided a high-resolution bandpass of approximately 0.7 eV at the Pd K-edge. The incident beam on the sample had a spot size of 200 × 100 μm^2. Pd K-edge (~24.35 keV) HERFD-XANES was measured with a 5-crystal Johann-type spectrometer equipped with Ge(11 11 11) analyzer crystals (Bragg angle: 80.3° at the Pd Kα emission line) and a curvature radius of 1 m. Total fluorescence yield (TFY) mode was employed for EXAFS data acquisition, where the acquisition efficiency is needed over resolution. The sample was positioned at a 55° angle relative to the incident beam to optimize signal detection and allow for simultaneous fluorescence and high-energy-resolution measurements. For Ti K-edge measurements (~4.97 keV), HERFD-XAS (high energy resolution fluorescence detection) was used with a 5-crystal Johann-type spectrometer equipped with Ge(400) analyzer crystals (Bragg angle: 76.3° at the Ti Kα emission line) and a curvature radius of 1 m. During the measurements, the electrochemical procedure and XAS acquisition were initiated simultaneously. Each sequence consisted of two preliminary d-scans followed by XANES/EXAFS scans. To ensure data consistency, the two d-scans as well as the first and last XANES/EXAFS scans were excluded from analysis. The spectra reported in this study were therefore acquired after two d-scans and one XANES/EXAFS scan, approximately 3–5 minutes after the start of electrochemical operation, reflecting quasi-steady-state conditions. To minimize the beam-induced damage to the membrane during operando XAS measurement, the X-ray beam was periodically moved to fresh positions on the electrode, and the dwell time per scan was kept as short as practical to limit the total dose at any single spot.

5. Supplementary Figures

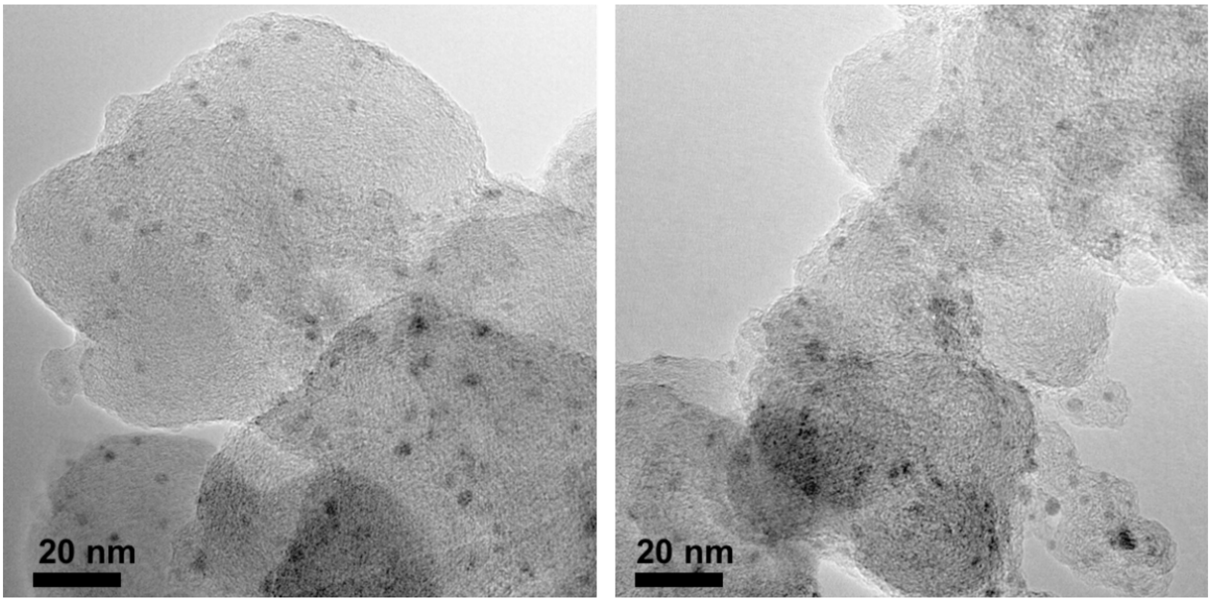

Figure S1. TEMs for the Pd/C sample.

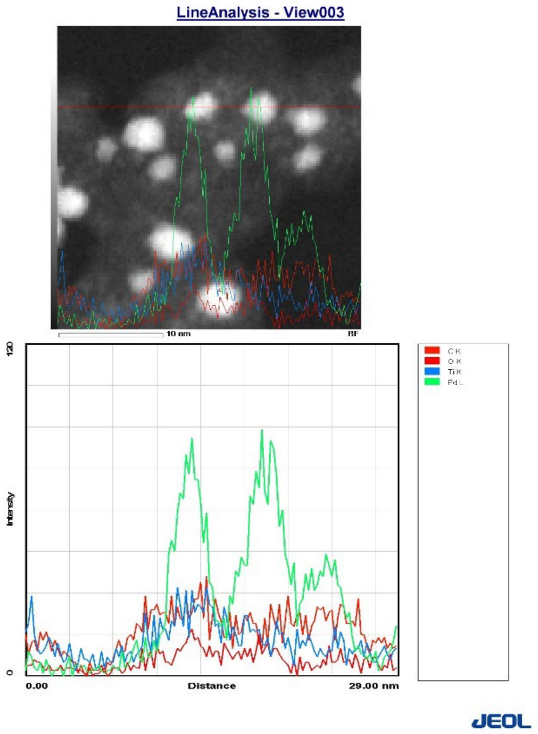

Figure S2. EDS line scan profile for the 24-cycle Pd@TiO_2/C sample. Red C and O, blue Ti, green Pd.

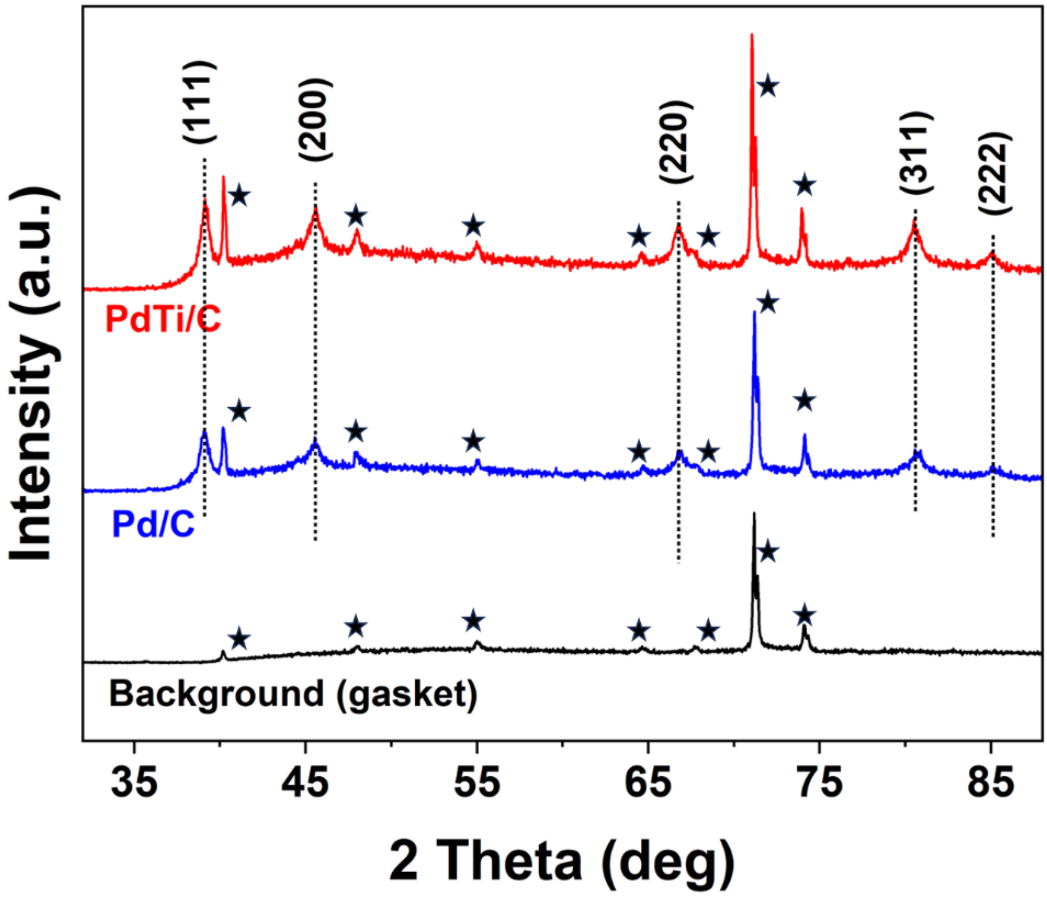

Figure S3. XRD pattern for the Pd/C and Pd@TiO_2/C samples.

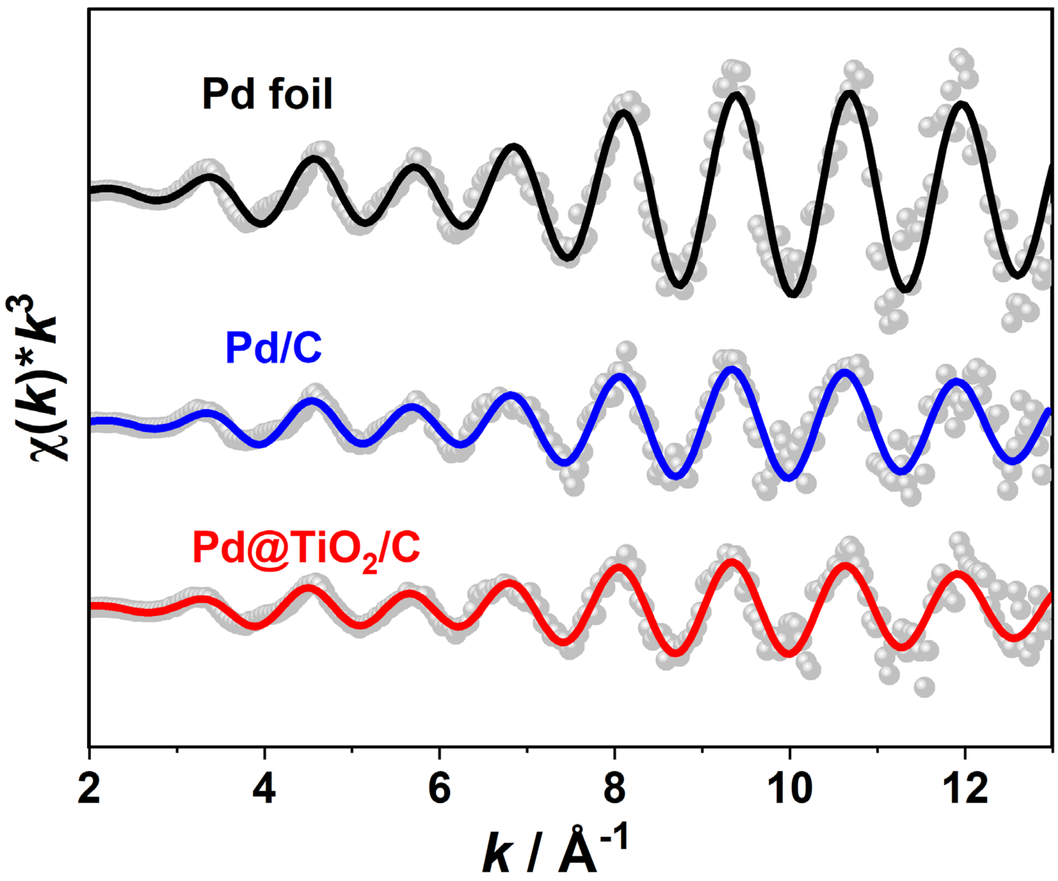

Figure S4. The Pd K-edge EXAFS for the Pd foil, Pd/C and Pd@TiO_2/C, shown in k^3 weighted k-space.

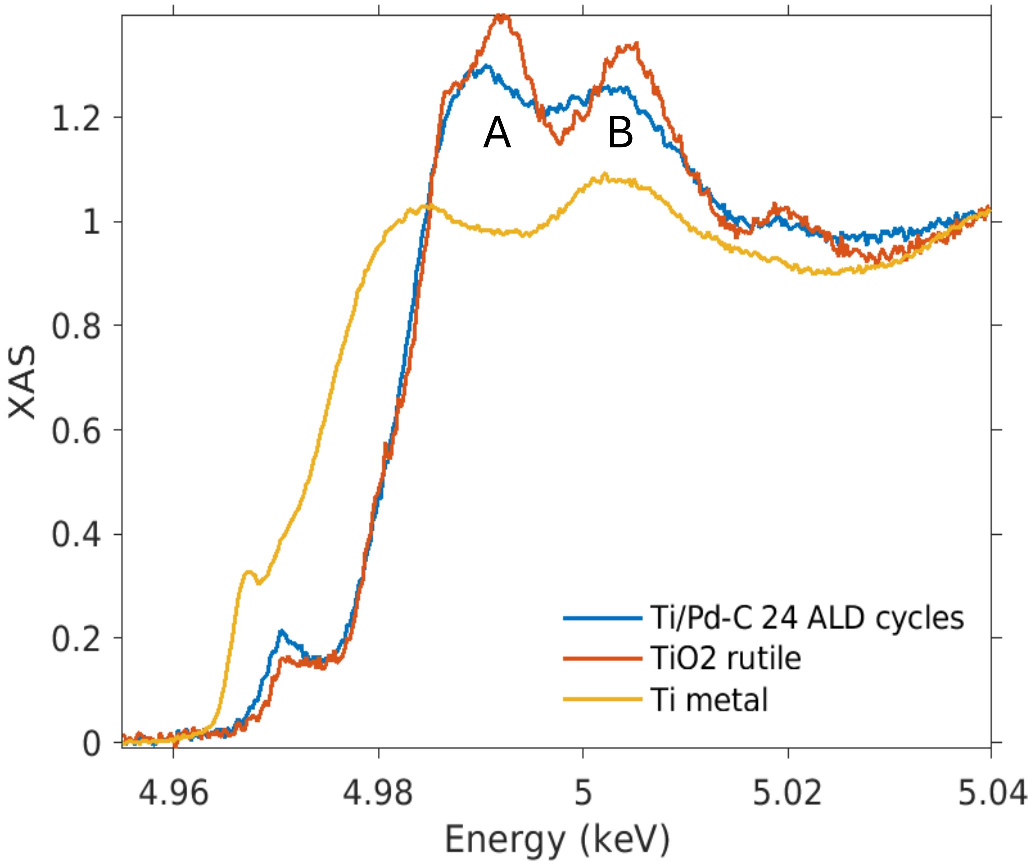

Figure S5. Lab-scale Ti K-edge XANES for Pd@TiO_2/C, TiO_2 rutile reference and Ti metal.

Lab-scale XANES were employed to compare the features of the Ti K-edge XANES for Pd@TiO_2/C to a TiO_2 rutile reference.

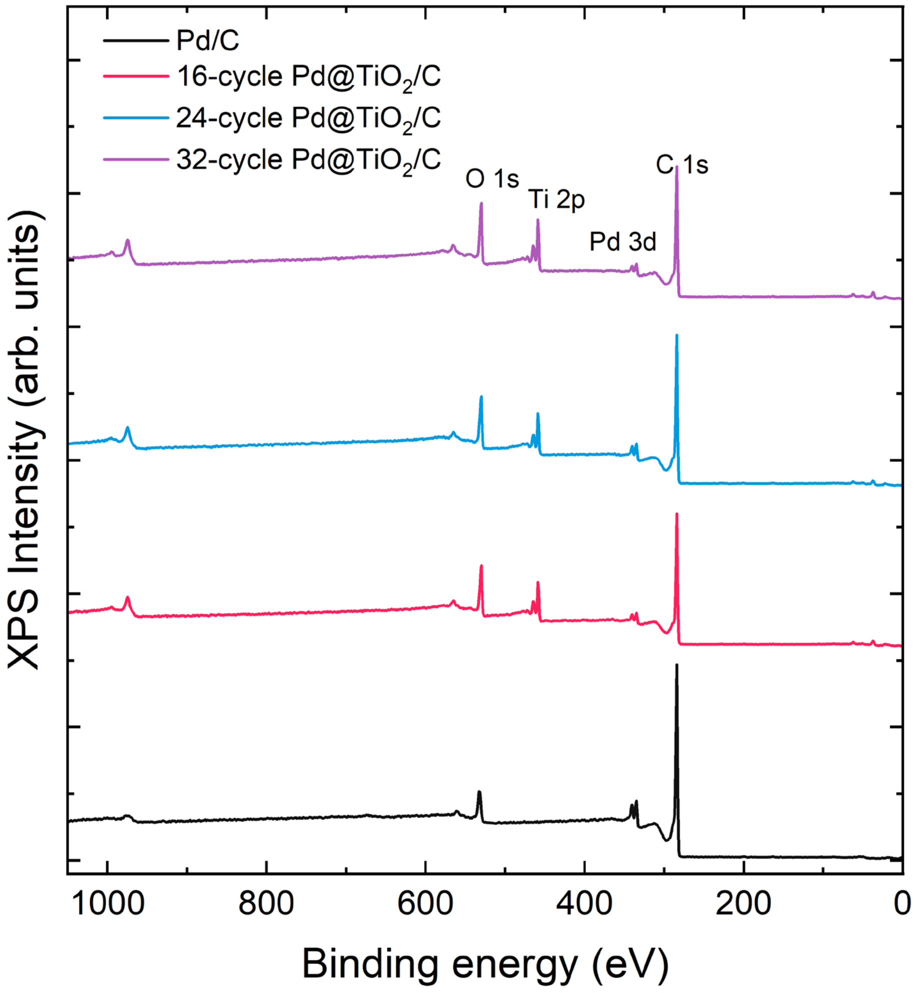

Figure S6. XPS survey spectra for Pd/C and $Pd@TiO_2/C$ with 16-, 24-, and 32-cycles ALD.

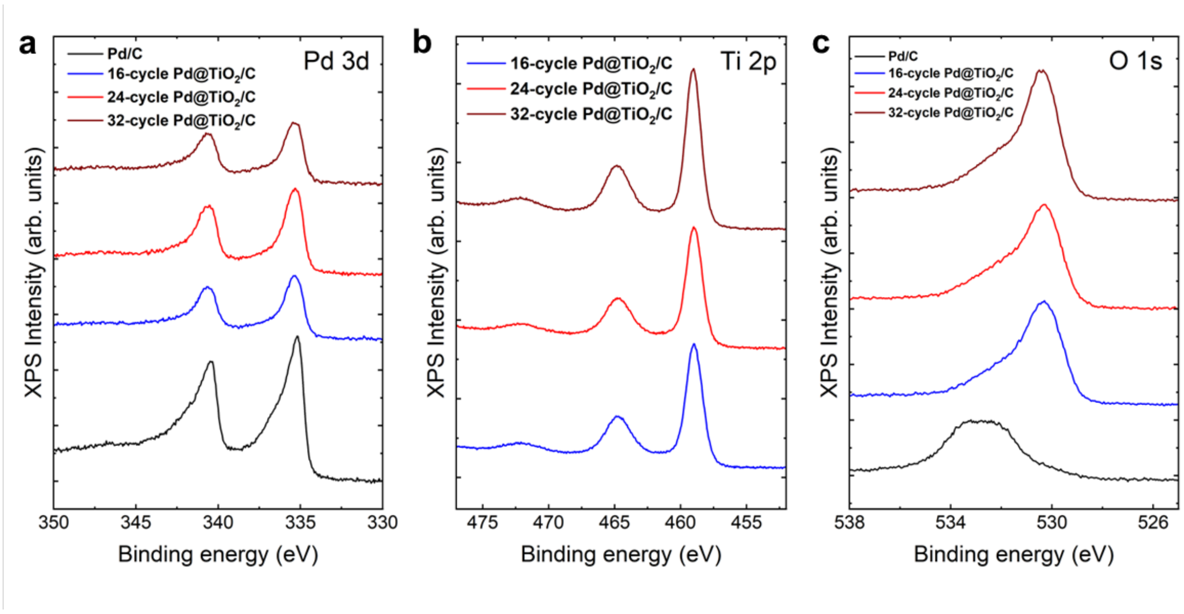

Figure S7. a) XPS of the Pd 3d, **b)** Ti 2p and **c)** O 1s region for the Pd/C and Pd@TiO_2/C samples with 16-, 24-, and 32-cycles ALD.

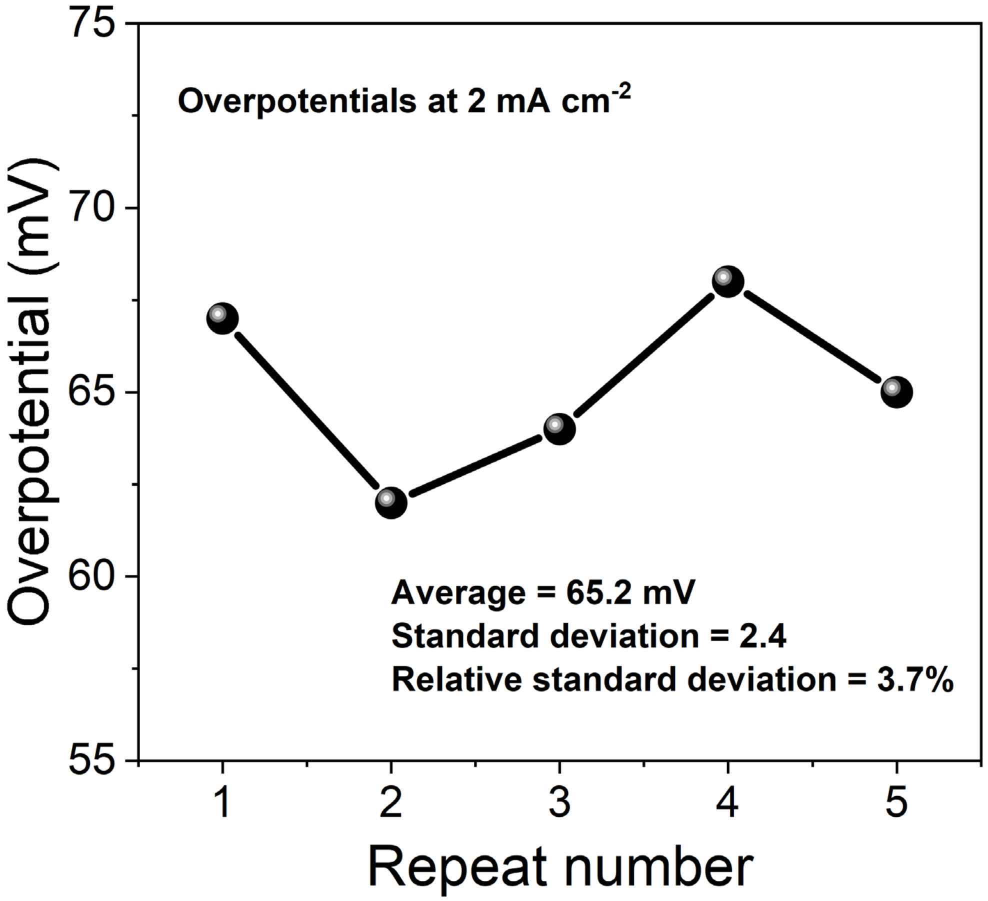

Figure S8. Repeatability of the overpotentials at 2 mA cm^{-2} for 24-cycle Pd@TiO_2/C catalysts in HOR.

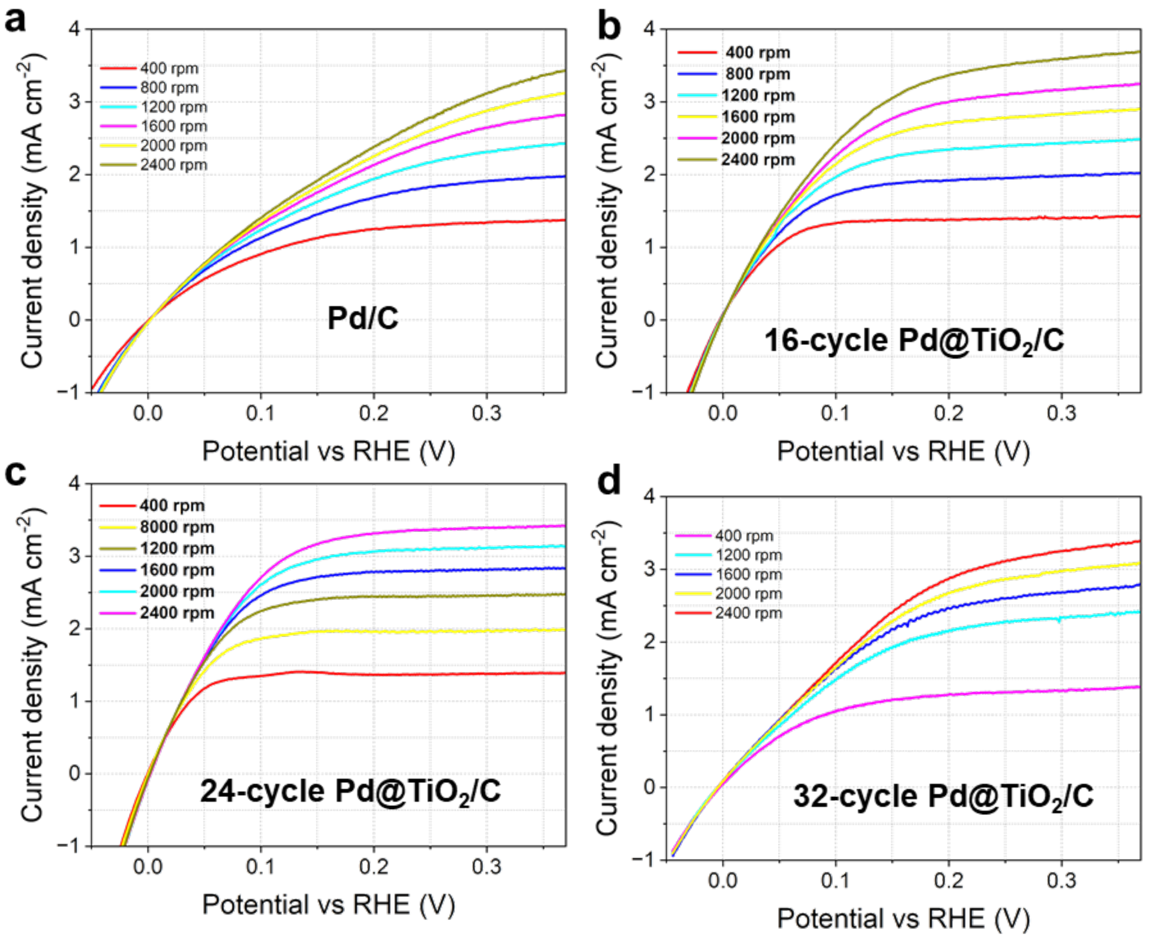

Figure S9. LSVs for **a)** Pd/C, **b)** 16-cycle Pd@TiO_2/C, **c)** 24-cycle Pd@TiO_2/C, and **d)** 32-cycle Pd@TiO_2/C at scan rate of 400, 800, 1200, 1600, 2000, and 2400 rpm.

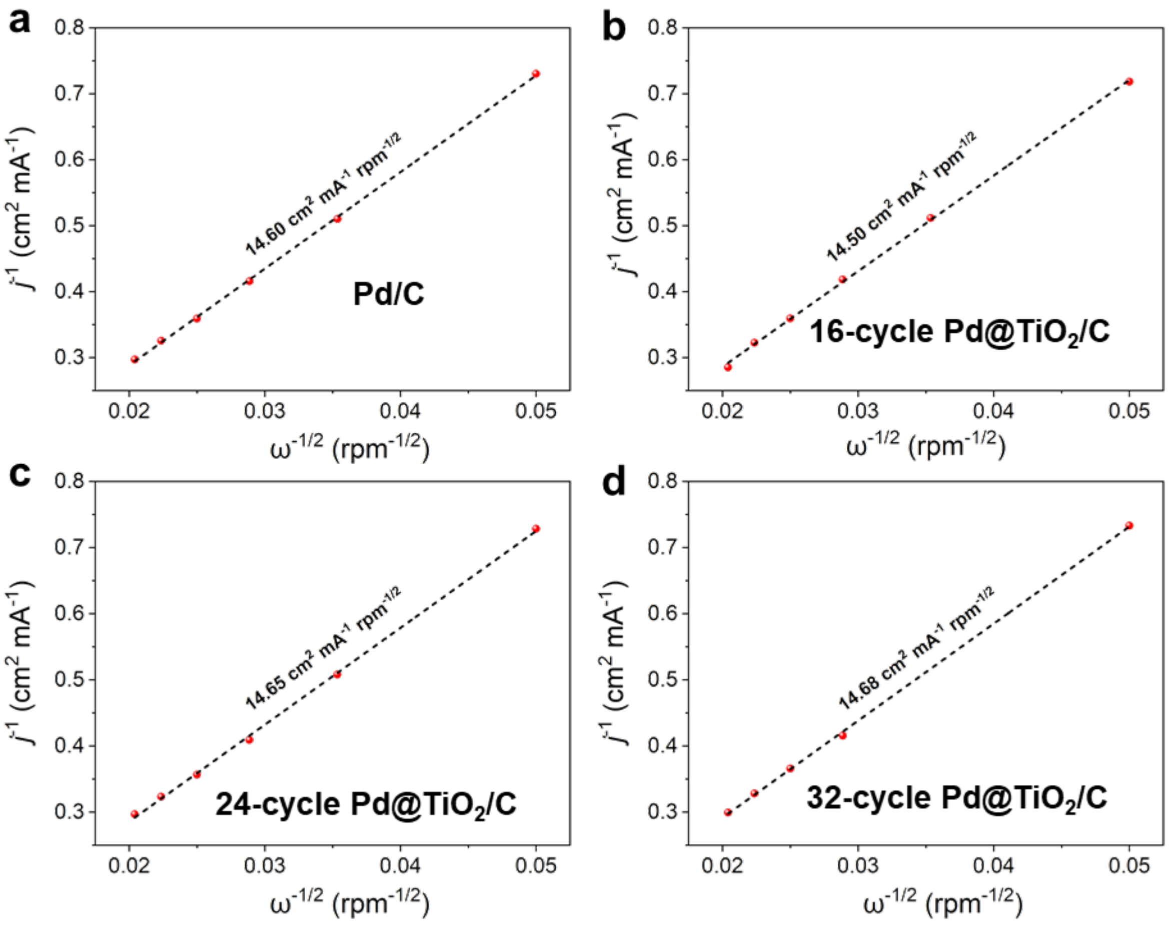

Figure S10. Koutecky-Levich plots for **a)** Pd/C, **b)** 16-cycle Pd@TiO_2/C, **c)** 24-cycle Pd@TiO_2/C, and **d)** 32-cycle Pd@TiO_2/C.

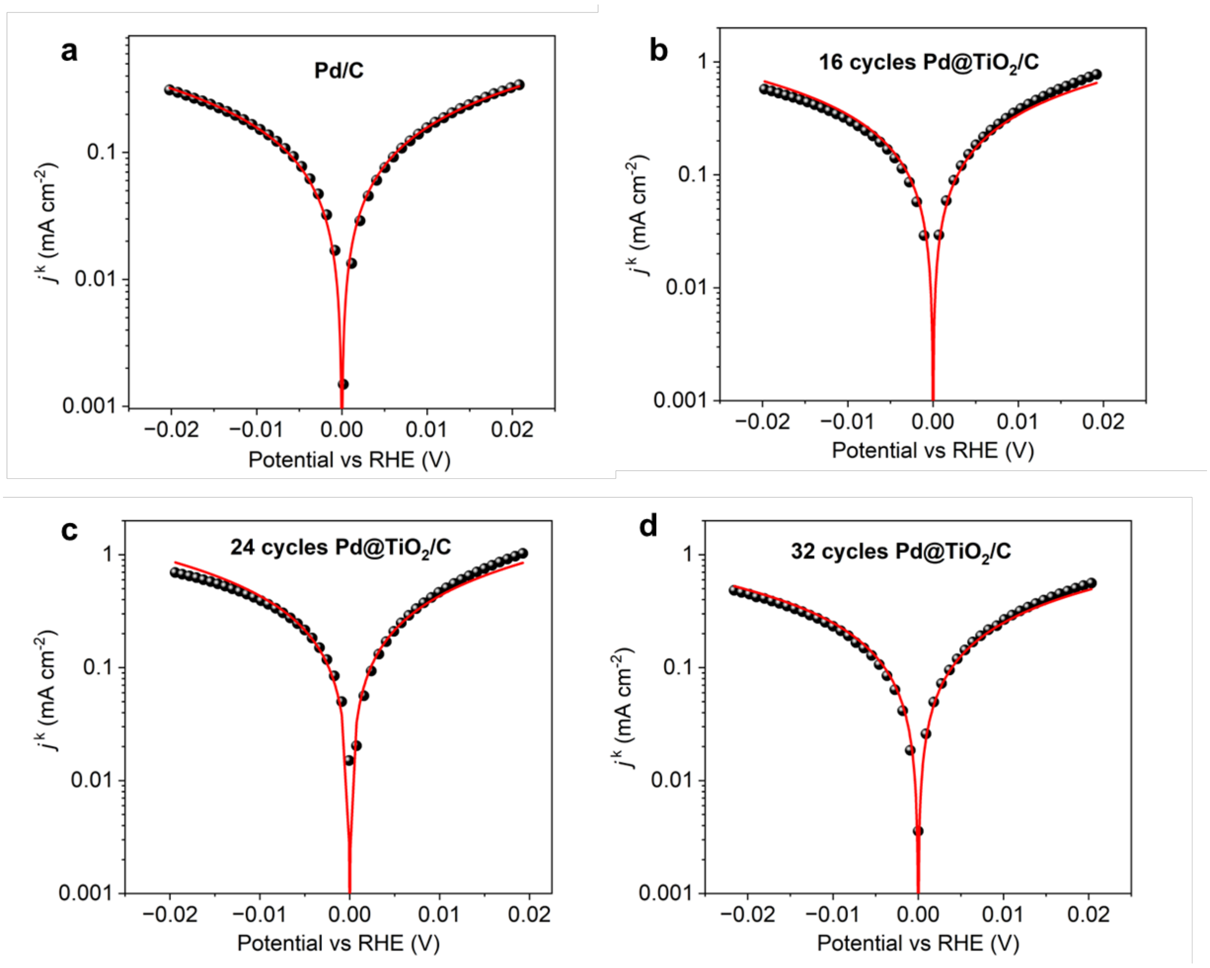

Figure S11. Non-linear fitting of the Tafel plots for **a)** Pd/C, **b)** 16-cycle Pd@TiO_2/C, **c)** 24-cycle Pd@TiO_2/C, and **d)** 32-cycle Pd@TiO_2/C.

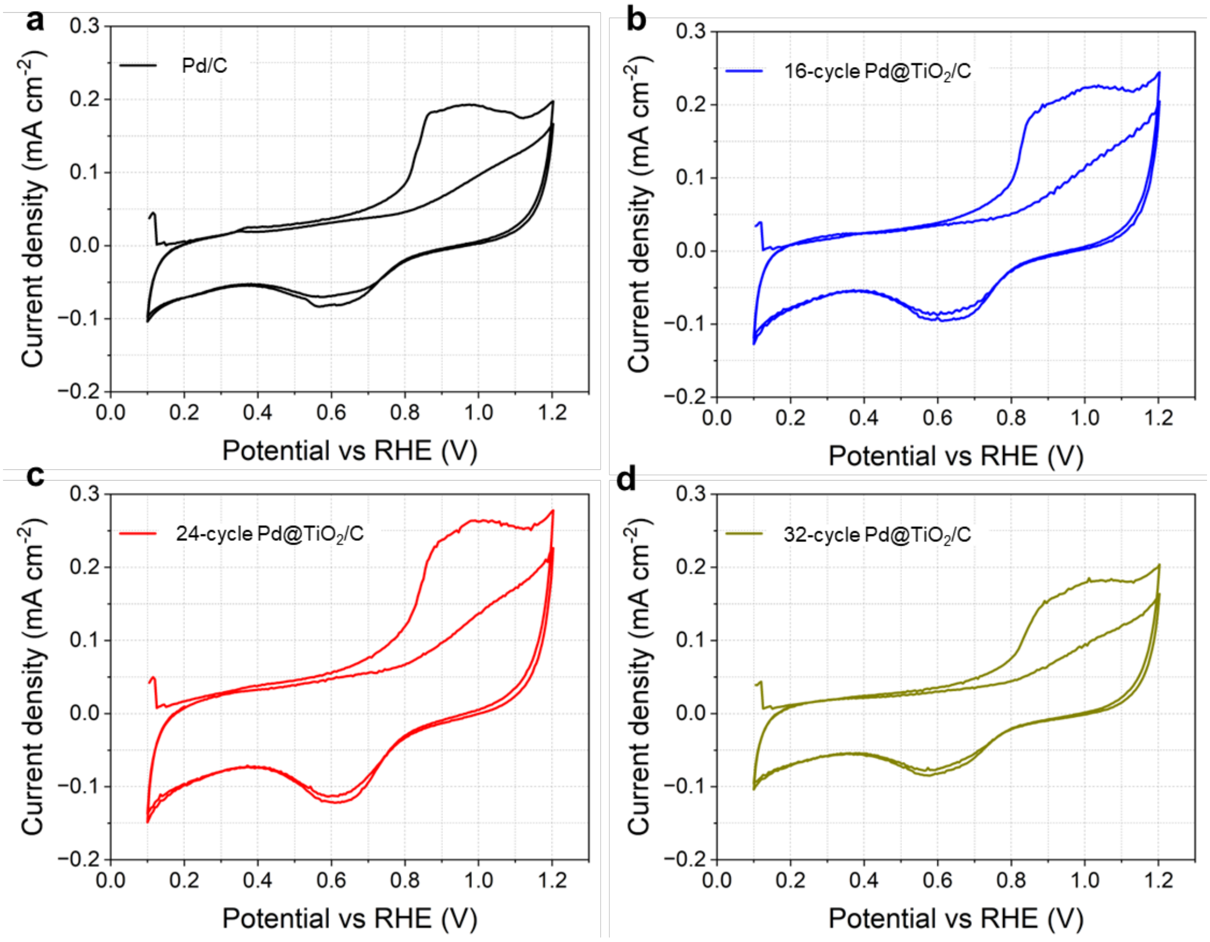

Figure S12. CO stripping for **a)** Pd/C, **b)** 16-cycle Pd@TiO_2/C, **c)** 24-cycle Pd@TiO_2/C, and **d)** 32-cycle Pd@TiO_2/C.

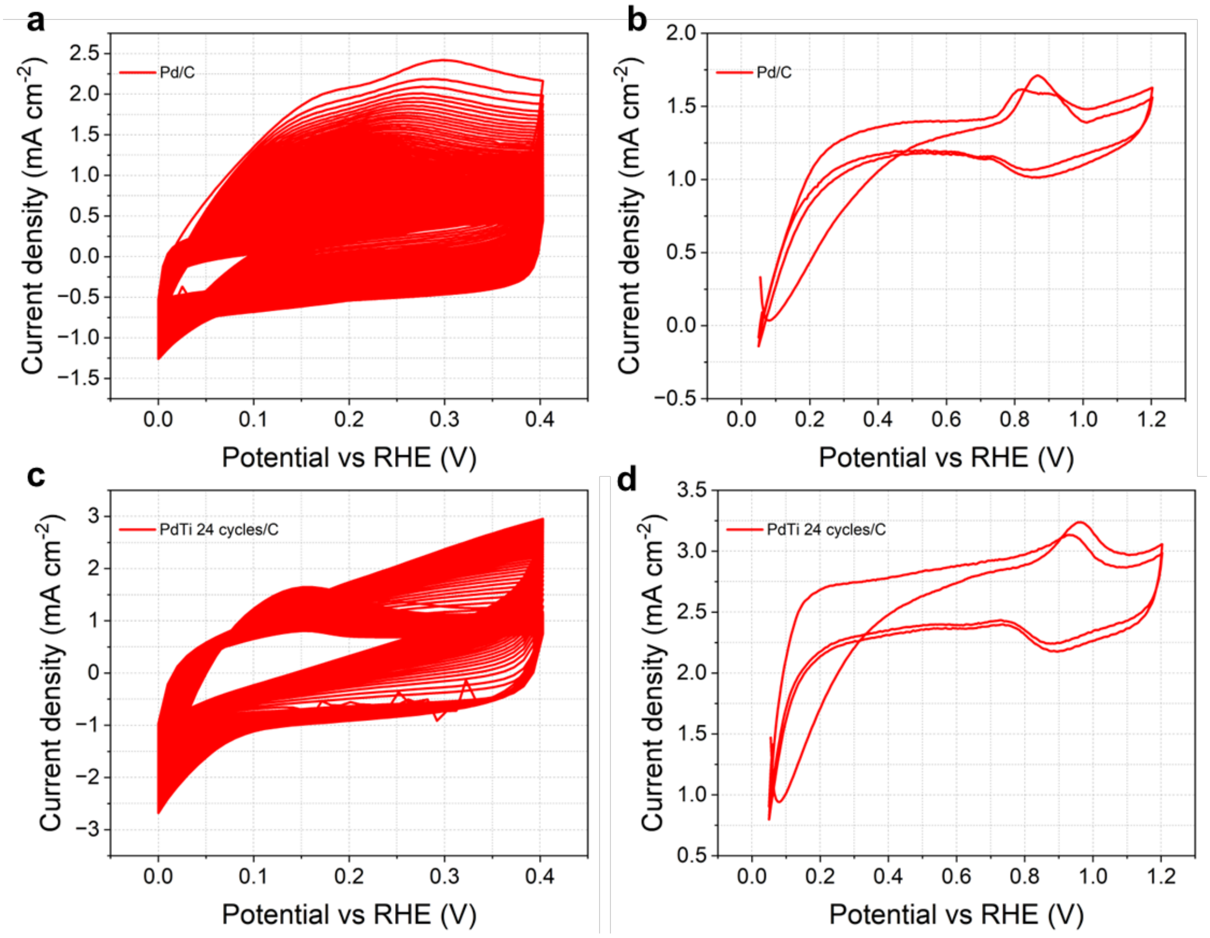

Figure S13. a) 7 000 CV cycles at a scan rate of 100 mV s^{-1} and **b)** 2 CV cycles at 20 mV s^{-1} for Pd/C in H_2-saturated 0.1 M KOH. **c)** 7 000 CV cycles a scan rate of 100 mV s^{-1} and **d)** 2 CV cycles at 20 mV s^{-1} for the 24-cycle Pd@TiO_2/C sample in H_2-saturated 0.1 M KOH.

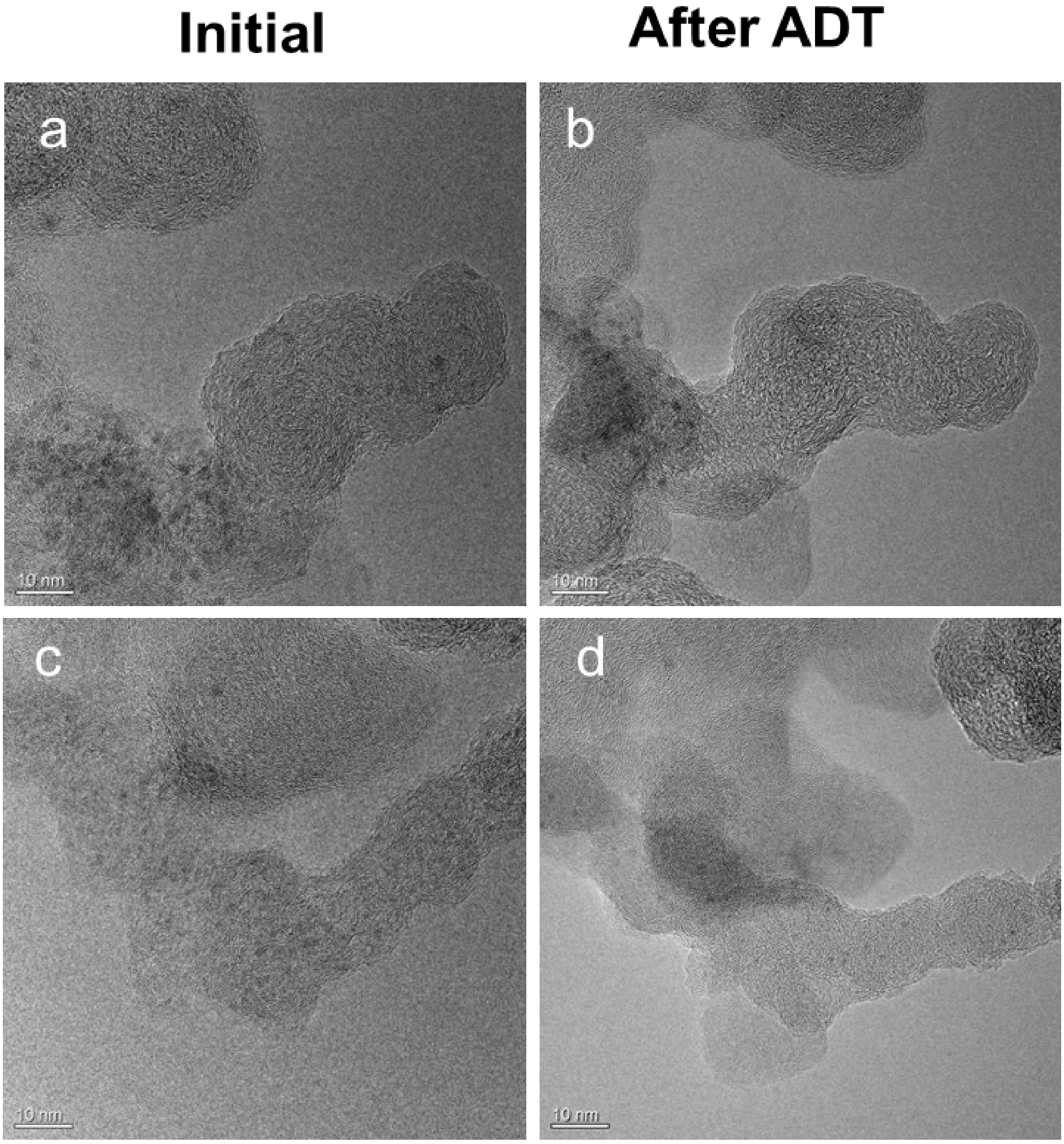

Figure S14. a) and **b)** IL-TEM images for the Pd/C sample with 5 wt% Pd loading. c) and d) IL-TEM images for the 24-cycle Pd@TiO_2/C sample with 5 wt% Pd loading.

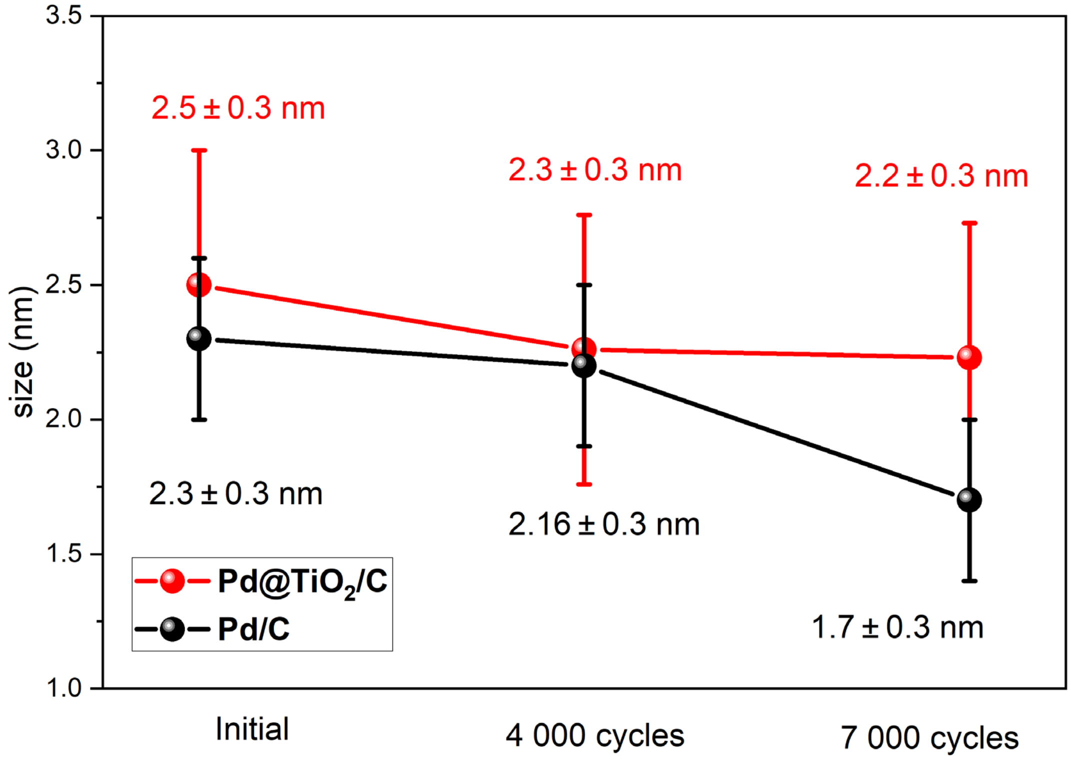

Figure S15. Size distribution for Pd/C and 24-cycle Pd@TiO_2/C at the initial state, after 4 000 cycles and after 7 000 cycles.

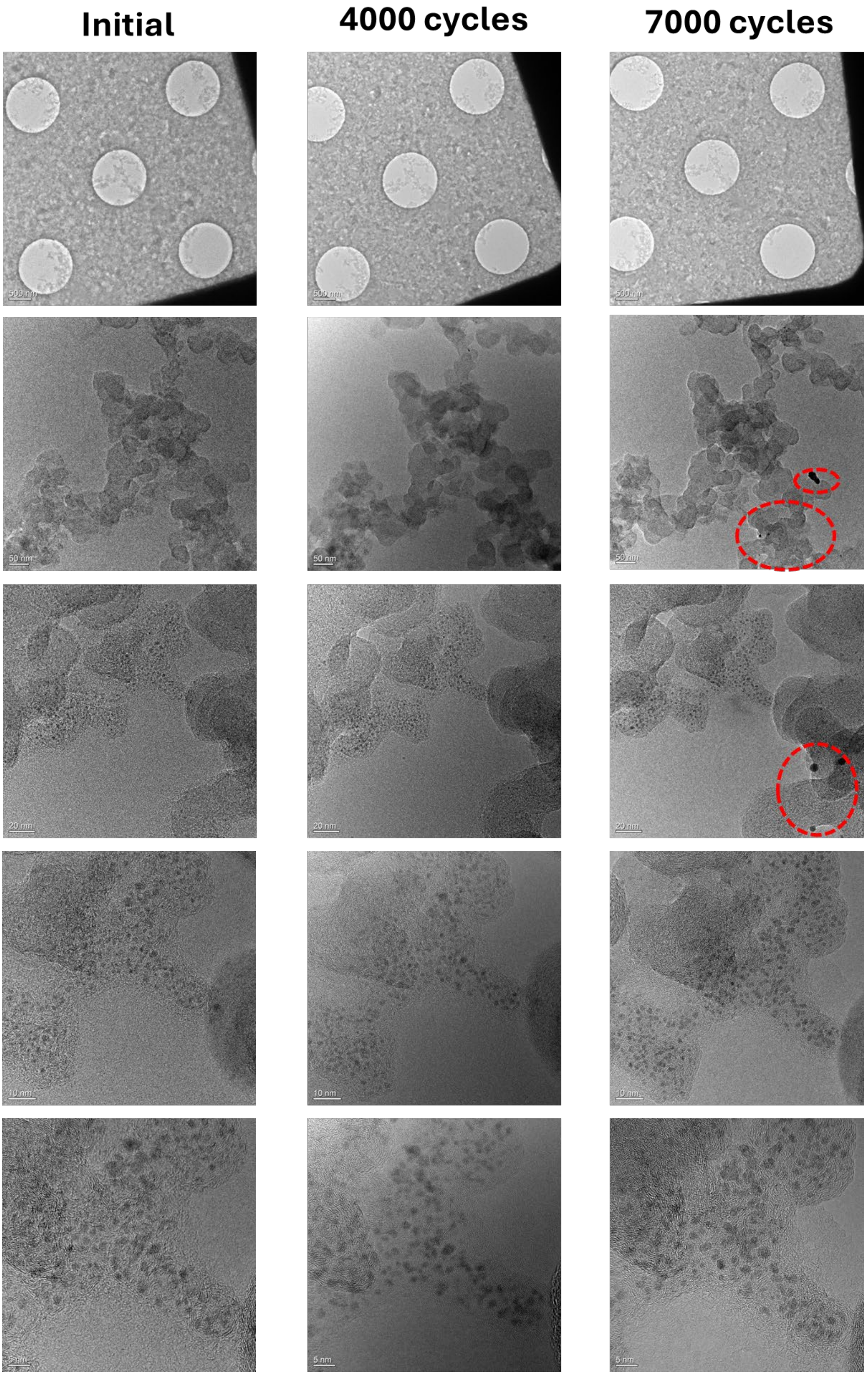

Figure S16. IL-TEM images for 24-cycle Pd@TiO_2/C.

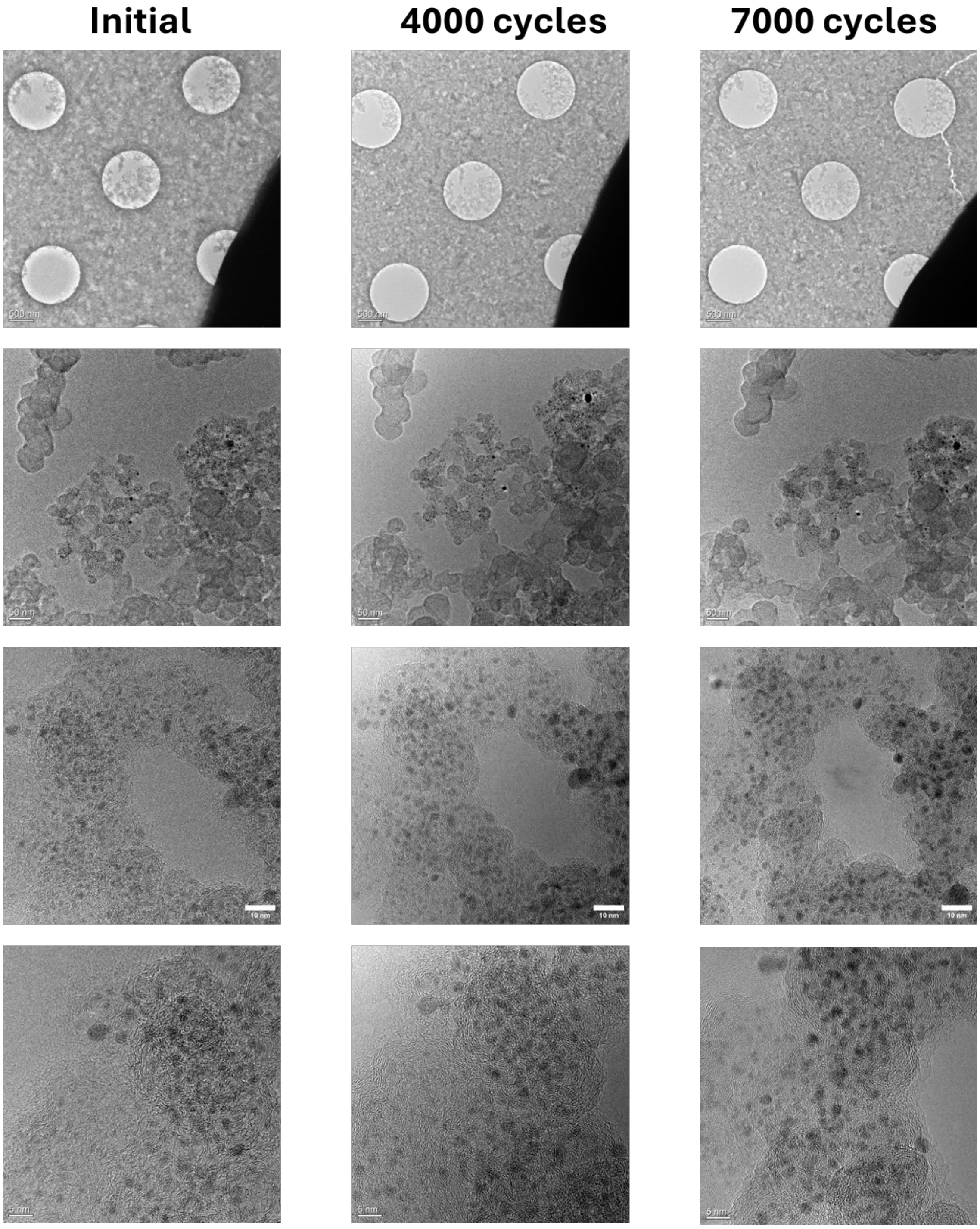

Figure S17. IL-TEM images for 24-cycle $Pd@TiO_2/C$.

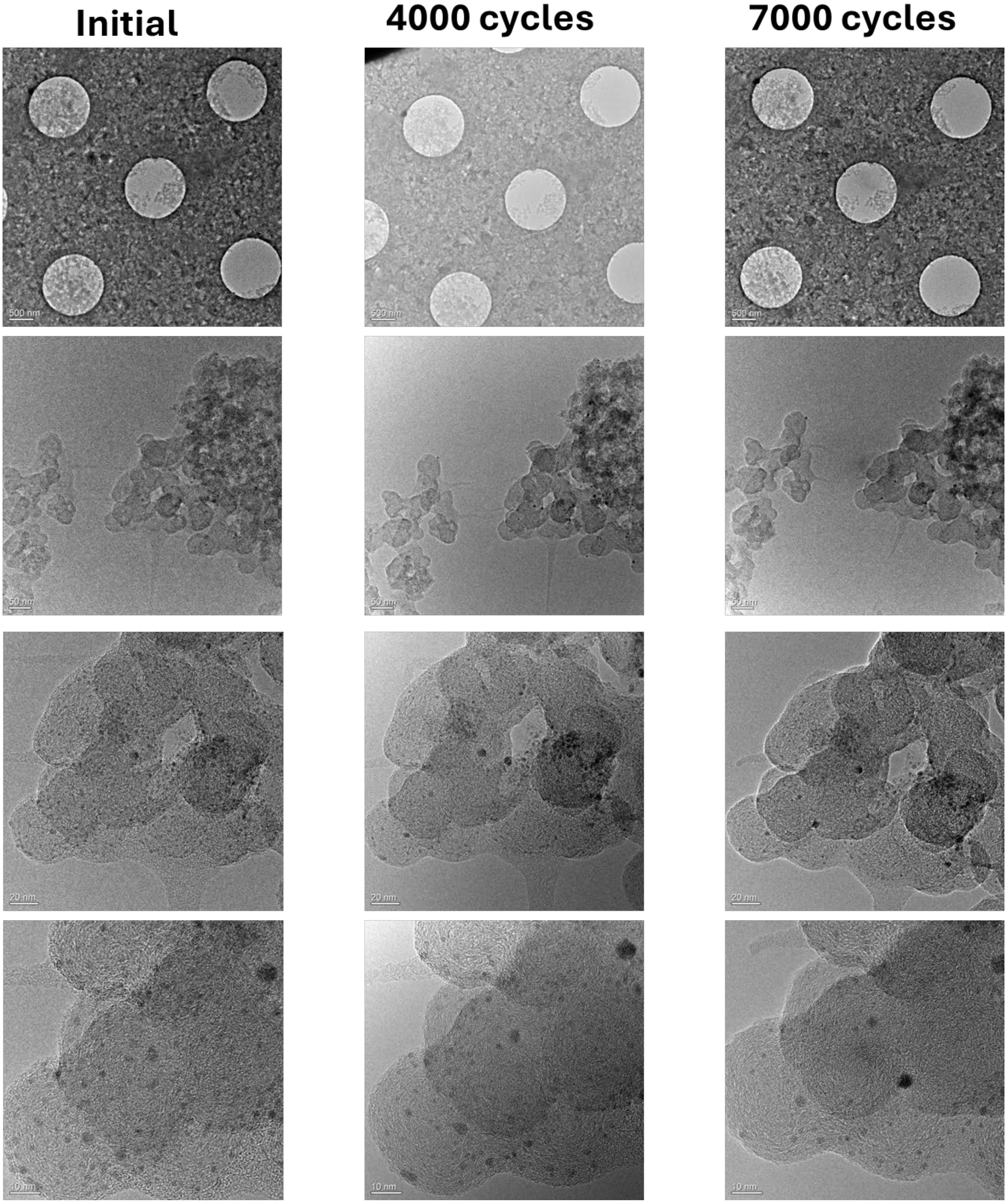

Figure S18. IL-TEM images for 24-cycle Pd@TiO_2/C.

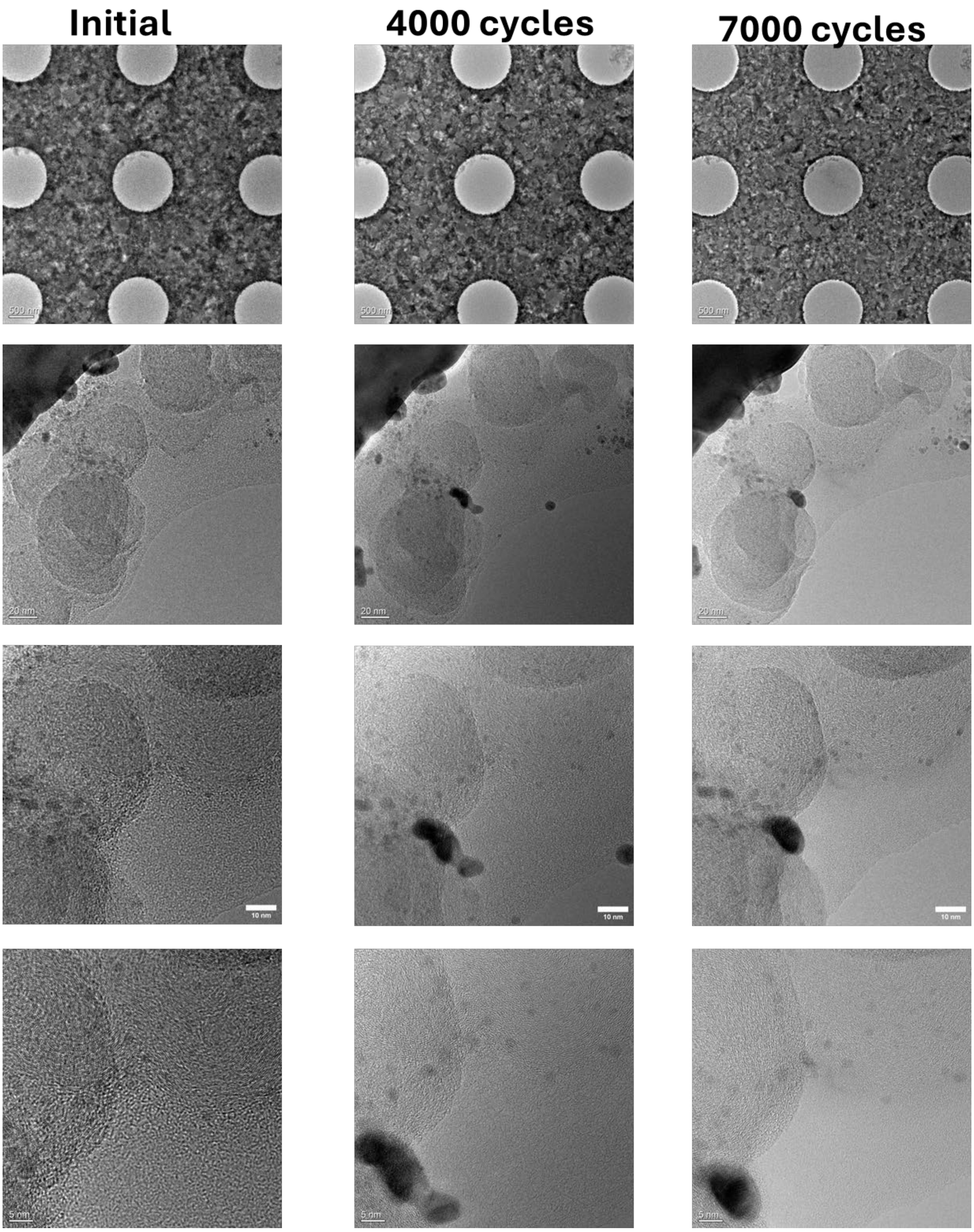

Figure S19. IL-TEM images for Pd/C sample.

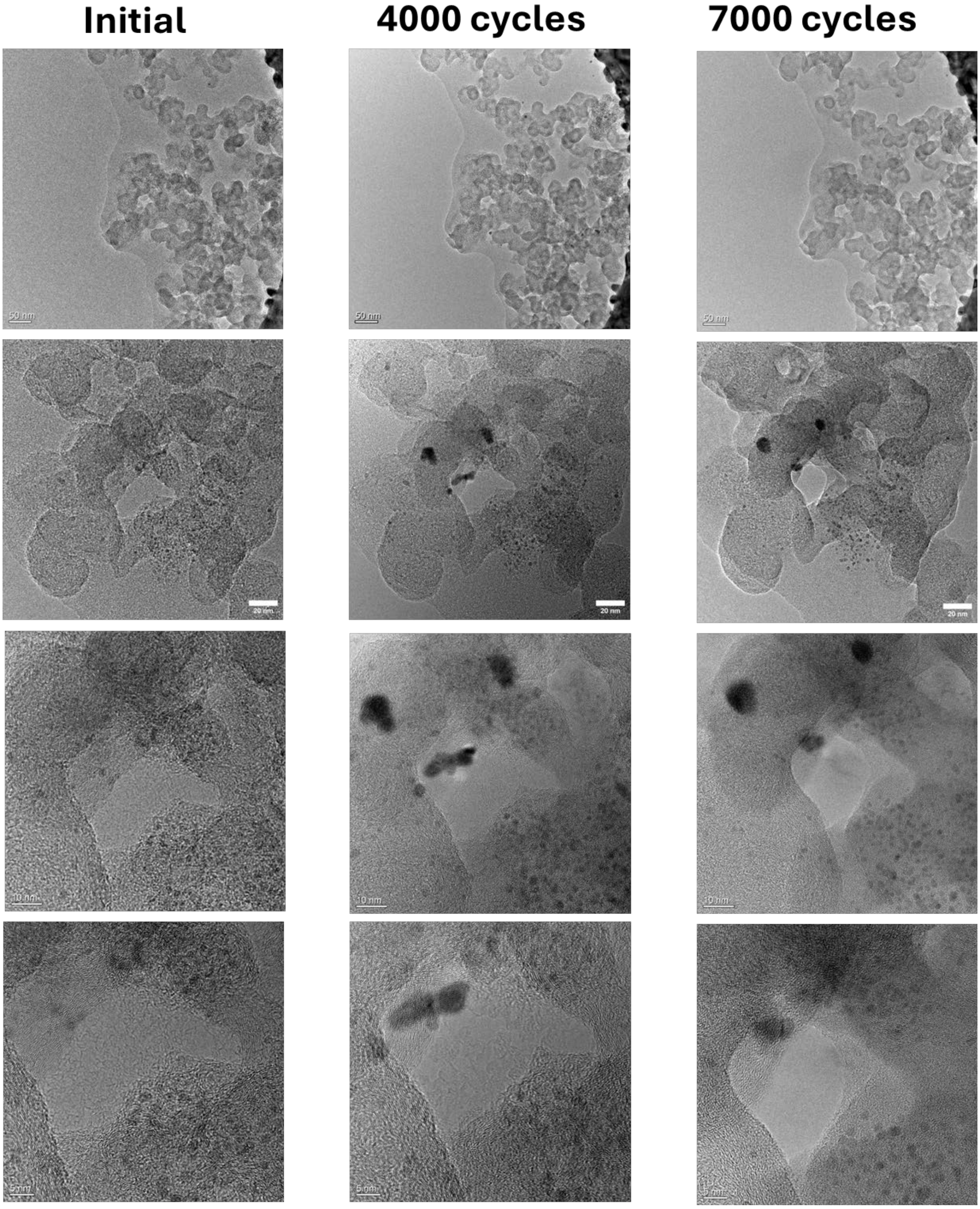

Figure S20. IL-TEM images for Pd/C sample.

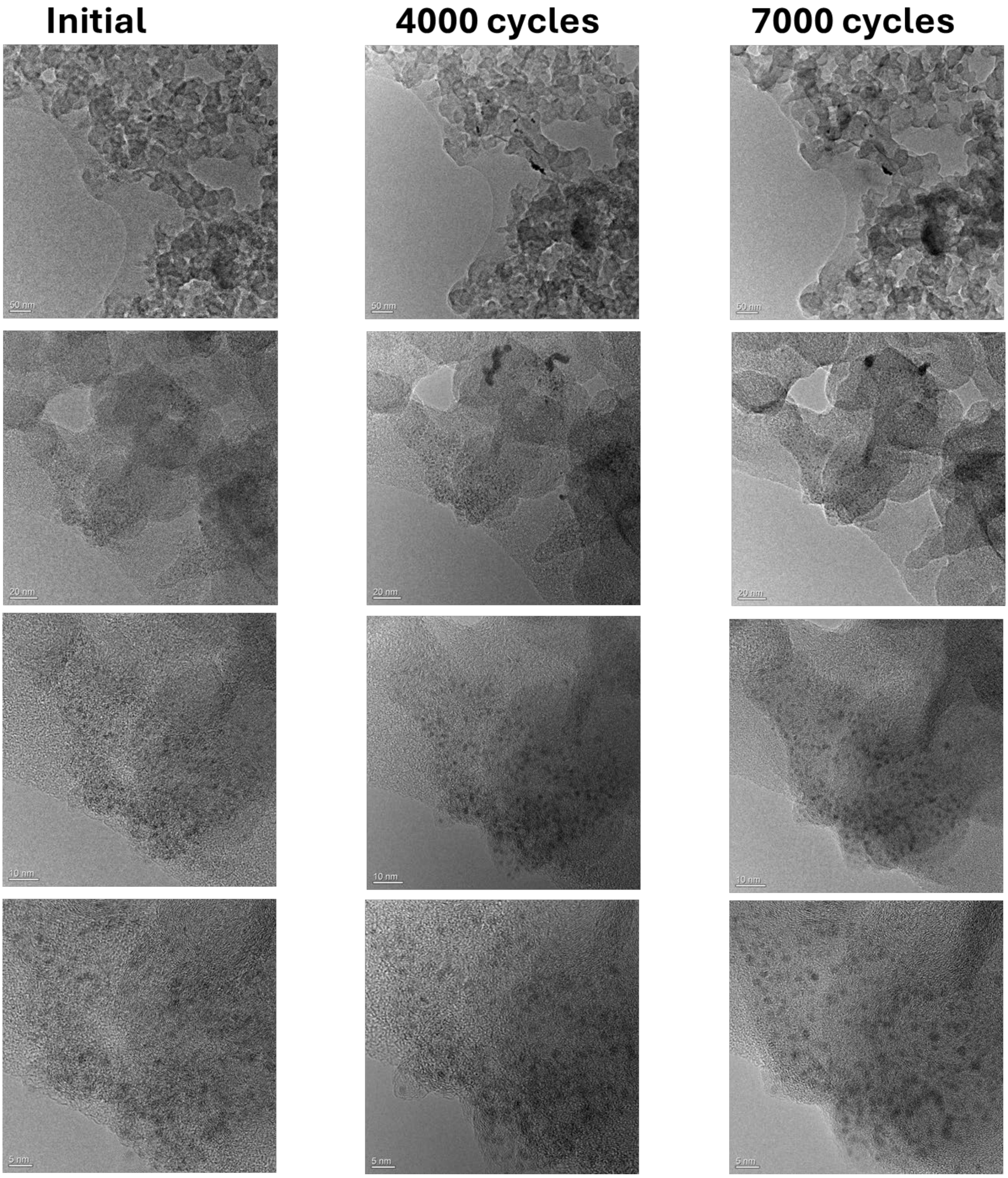

Figure S21. IL-TEM images for Pd/C sample.

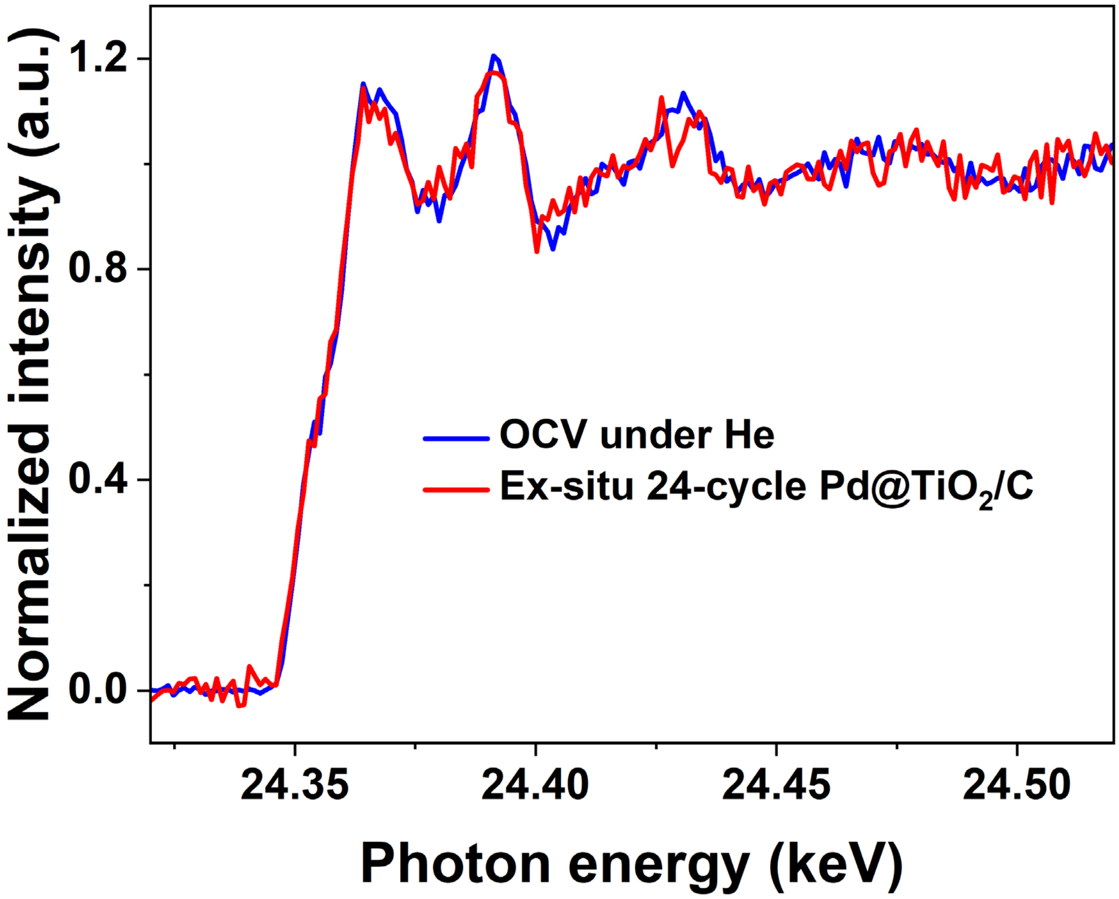

Figure S22. The operando (OCV in He) and ex situ XANES of Pd@TiO_2/C.

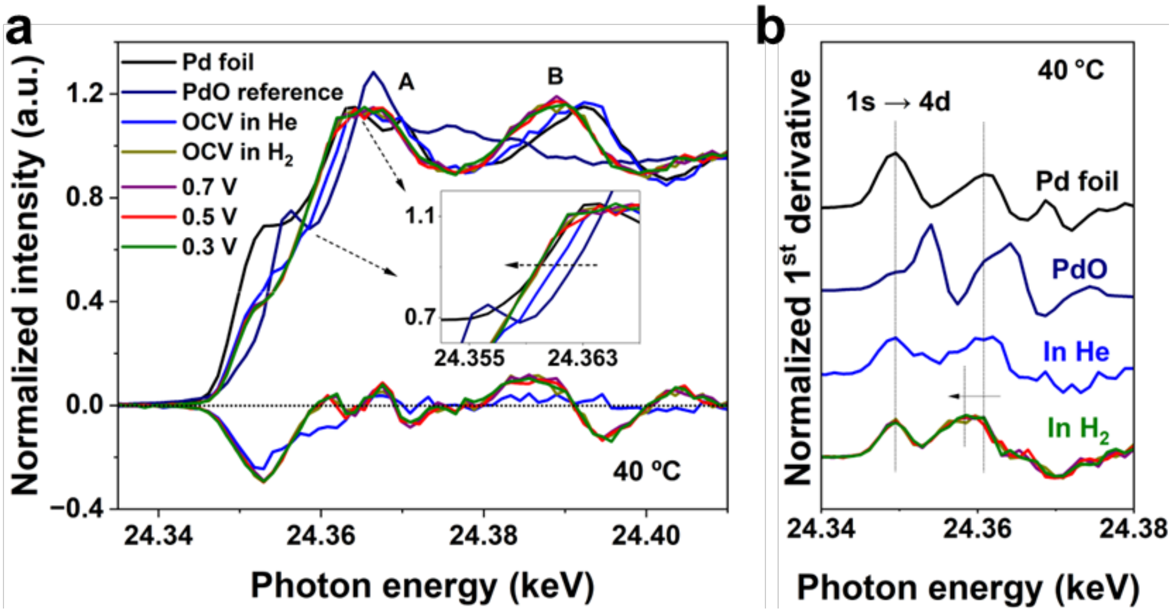

Figure S23. **a)** Normalized Pd K-edge XANES spectra of Pd@TiO_2/C under OCV in He and H_2, and at applied voltages of 0.7 V, 0.5 V, and 0.3 V in H_2 at 40 °C; difference spectra shown below. **b)** First derivative of the XANES spectra in **a)**.

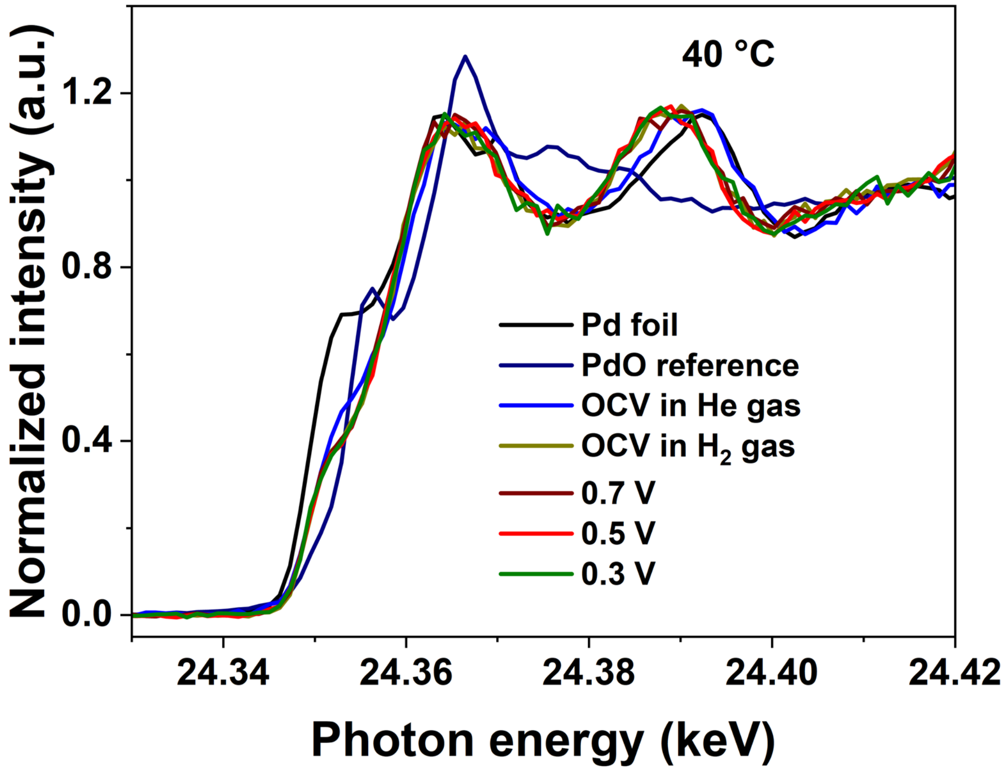

Figure S24. Normalized Pd K-edge XANES spectra of Pd /C under OCV in He and H_2, and at applied voltages of 0.7 V, 0.5 V, and 0.3 V in H_2 at room temperature.

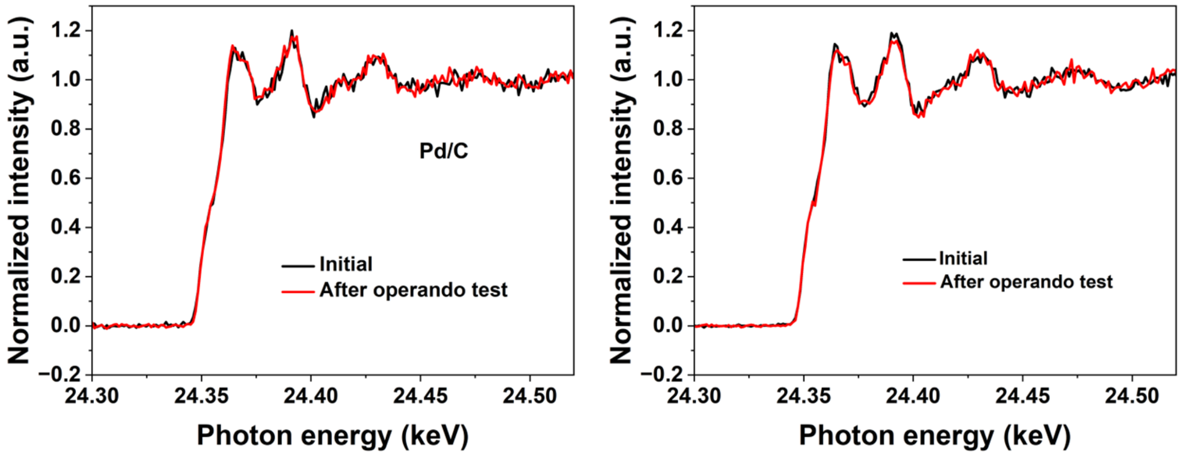

Figure S25. Comparison of Pd/C and Pd@TiO_2/C before and after operando XAS test.

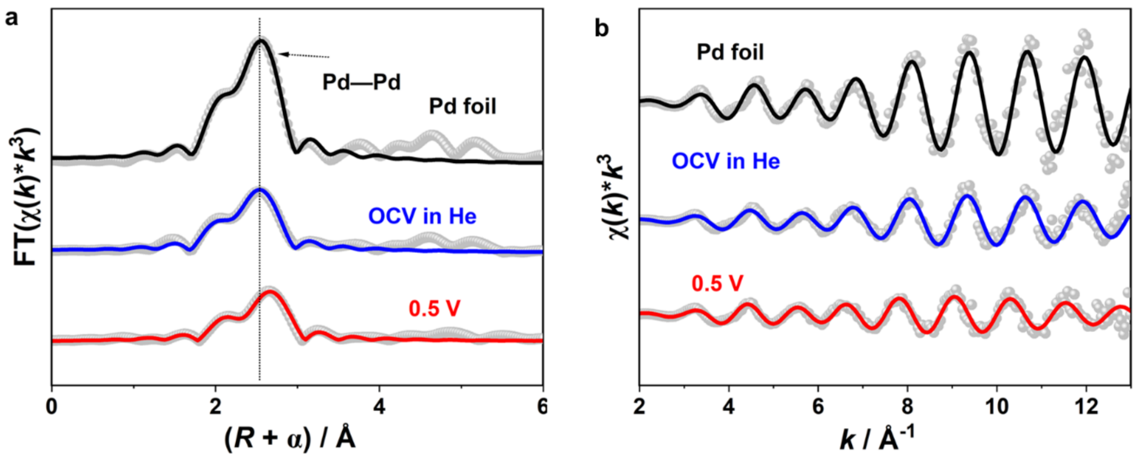

Figure S26. a) Fourier-transformed EXAFS spectra and corresponding fits, **b**) Pd K-edge EXAFS for the Pd foil and Pd/C under OCV in He and 0.5 V in H_2. Shown in k^3 weighted.

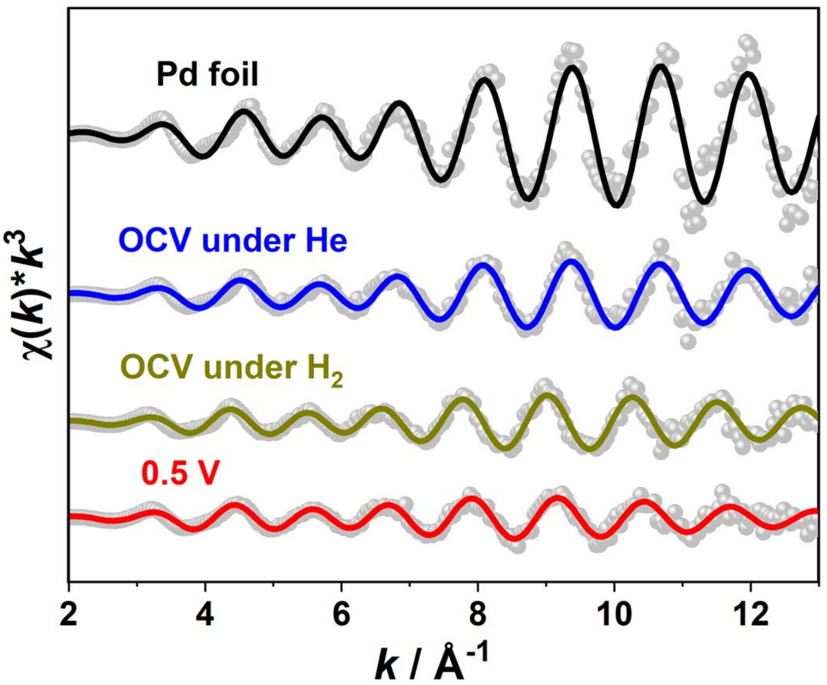

Figure S27. The Pd K-edge EXAFS for the Pd foil, Pd@TiO_2/C in OCV under He, Pd@TiO_2/C in OCV under H_2, and Pd@TiO_2/C under 0.5 V, shown in k^3 weighted k-space.

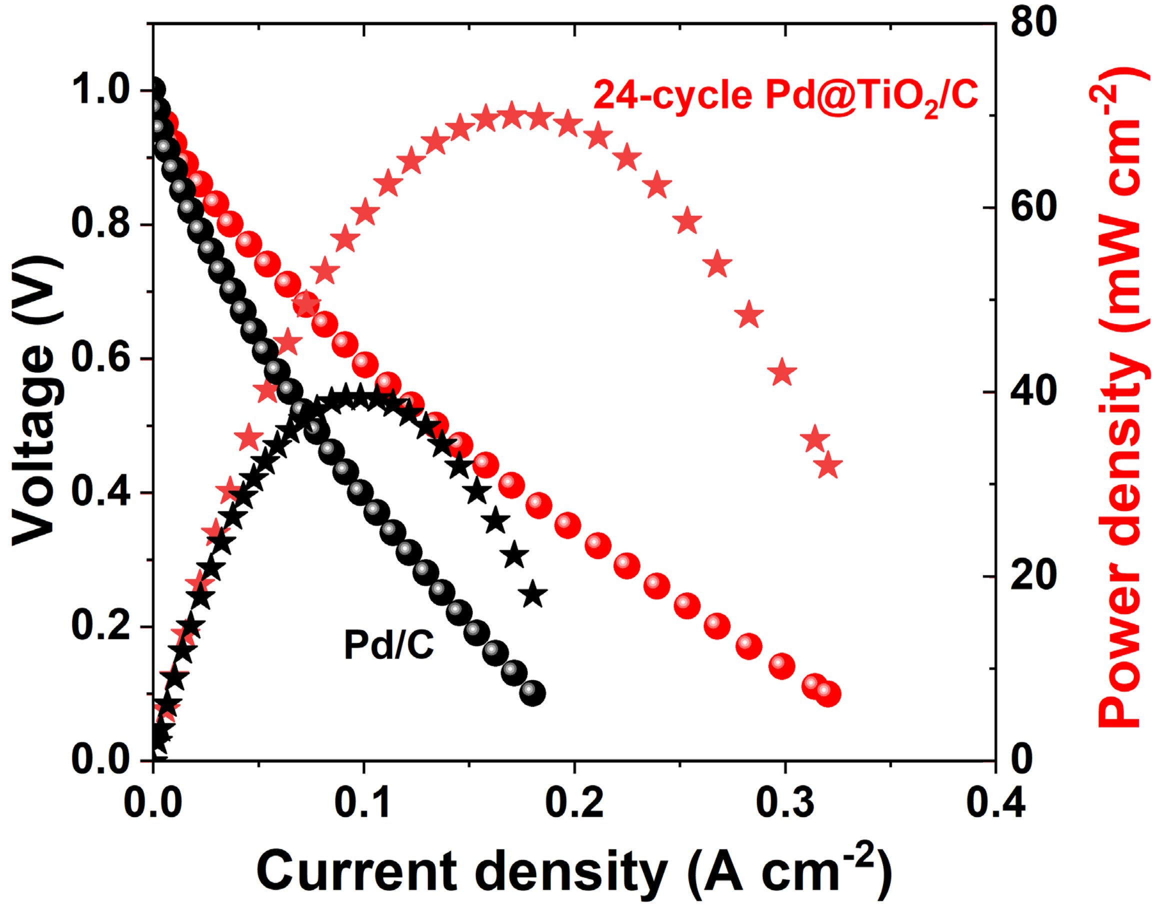

Figure S28. The AEMFC performance of Pd/C and Pd@TiO_2/C at 40 °C.

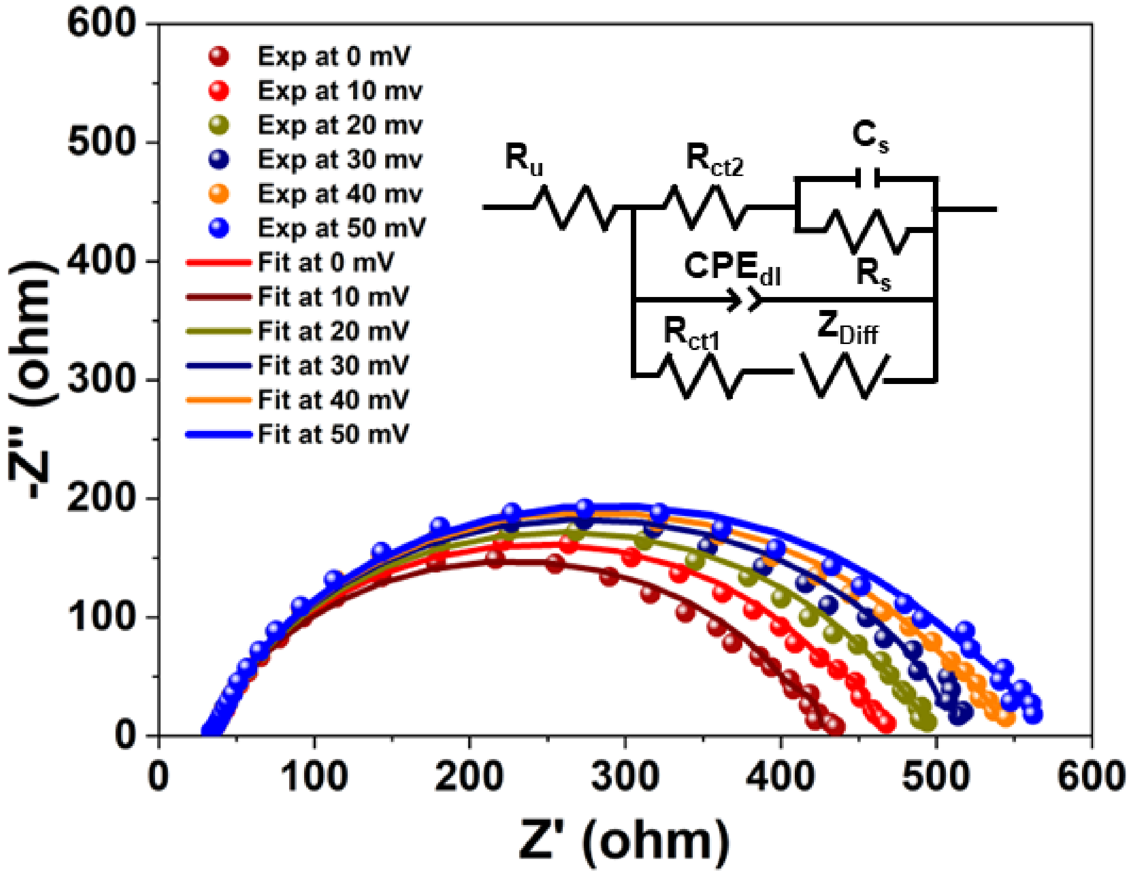

Figure S29. EIS data and the fitting results at different potentials for the Pd/C samples.

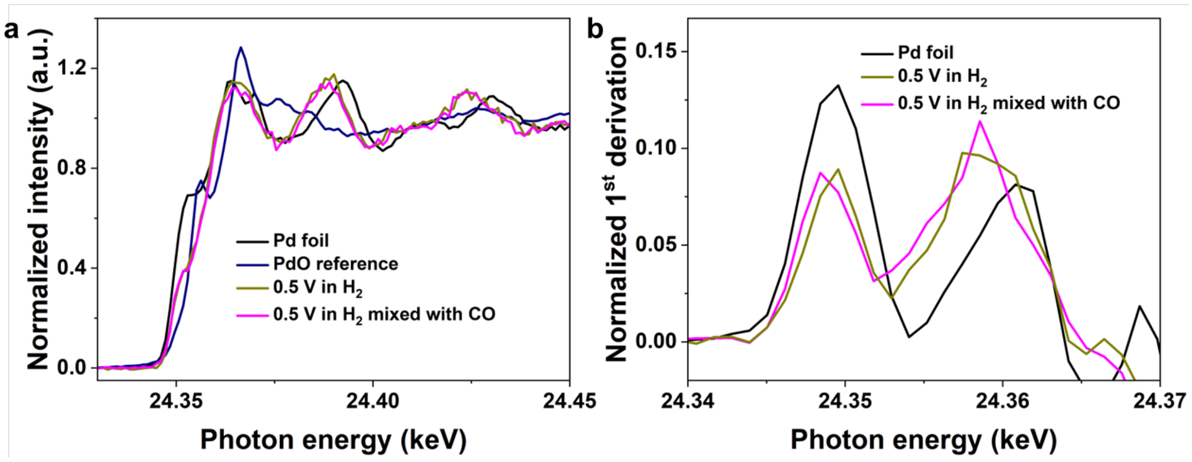

Figure S30. Operando XAS characterization of Pd/C under CO exposure. **a)** Normalized Pd K-edge XANES spectra measured at 0.5 V in H_2, 0.5 V in mixed H_2/CO (10 mL/min H_2, 0.02 mL/min CO) the Pd foil, and PdO references. **b)** First derivative of the XANES spectra in **a)**.

6. Supplementary Tables

Table S1. Curve fit Parameters for the Pd K-edge EXAFS.

**Samples**	**shell**	*N*[a]	**R(Å)**[b]	**σ^2 /Å^2**[c]	**ΔE0 (eV)**[d]	**R factor**[e]
Pd foil	Pd-Pd	12	2.734	0.0040	5.24	0.0034
Pd@TiO_2/C OCV under He	Pd-Pd	8.2 ± 0.6	2.734 ± 0.004	0.0066	3.51	0.0065
Pd/C OCV under He	Pd-Pd	7.7 ± 0.7	2.736 ± 0.005	0.0063	2.07	0.0117
Pd@TiO_2/C OCV under H_2	Pd-Pd	8.5 ± 0.7	2.830 ± 0.004	0.0075	4.05	0.0081
Pd@TiO_2/C under 0.5V	Pd-Pd	8.3 ± 0.7	2.789 ± 0.004	0.0097	3.72	0.0048
Pd/C under 0.5V	Pd-Pd	9.2 ± 0.6	2.827 ± 0.004	0.0087	5.58	0.0062
Pd/C ex situ	Pd-Pd	8.7 ± 0.7	2.746 ± 0.004	0.0062	4.93	0.0082
Pd@TiO_2/C ex situ	Pd-Pd	8.0 ± 0.6	2.740 ± 0.004	0.0067	3.14	0.0082

[a]*N*: coordination numbers; [b]R: bond distance; [c]σ^2: Debye-Waller factors; [d]ΔE_0: the inner potential correction. [e]R factor: goodness of fit.

The spectra were first normalized using Athena, and shell fitting was subsequently performed with Artemis. Self-absorption correction has been done for the Pd foil data in Athena using the Troger algorithm. The Pd CIF standard file from Crystallography Open Database was used for fitting data as a FEFF input file. The χ (k) function was Fourier transformed using k^3-weighting, and all fittings were carried out in *R*-space. The coordination parameters of the samples were obtained by fitting the experimental spectra with theoretical amplitude. The amplitude reduction factor (S_0^2) was set to 0.995, based on fitting the Pd foil reference with the coordination number fixed at 12. The Debye–Waller factor (σ^2) was treated as a variable during fitting. Notably, σ^2 values for samples under H_2 were slightly higher than those under He, indicating increased structural disorder. The inner potential correction (ΔE_0) was individually optimized for each sample to obtain the best fit for bond distances (*R* values) and coordination numbers. In this work, we perform only a first shell data analysis.

Table S2. The HOR performance in 0.1 M KOH.

**Catalysts**	$j^{0,s}$ **(mA cm$_{ECSA}^{-2}$)**	$j^{0,m}$ **(mA mg$_{Pd}^{-1}$)**
Pd/C	0.089	28
16-cycle Pd@TiO_2/C	0.164	78
24-cycle Pd@TiO_2/C	0.193	98
32-cycle Pd@TiO_2/C	0.175	59

Table S3. Comparison of the HOR activity in 0.1 M KOH with Pd-based Catalysts.

Catalysts	$j^{0,s}$ (mA cm_{ECSA}^{-2})	$j^{0,m}$ (mA mg_{Pd}^{-1})	References
**Pd/C**	**0.056**	**30.5**	This work
**24-cycle Pd@TiO$_2$/C**	**0.193**	**97.5**	
Pd-Pd$_4$S/C	0.2246	97.22	*Adv. Funct. Mater.* **2022**, *32*, 2113047.[2]
Pd$_4$S/C	0.0878	37.35	
Pd/C	0.0482	19.39	
0.38 CeO$_x$-Pd/C	0.118	51.54	*Adv. Funct. Mater.* **2020**, *30*, 2002087.[3]
Pd/C	0.045	20.84	
Pd/C-CeO$_2$: 6 wt%	0.089	19	*Nano Energy* **2017**, *33*, 293–305.[4]
Pd/C-CeO$_2$:10 wt%Pd	0.055	24	
Pd/C-CeO$_2$:20 wt% Pd	0.083	11	
PdCu/C-200 °C	0.0156	11.2892	*J. Am. Chem. Soc.* **2018**, *140*, 16580–16588.[5]
PdCu/C-500 °C	0.2159	127.6172	
Pd/C	0.0607	37.5692	
Pd/C-CeO$_2$	0.055	24	*Angew. Chem. Int. Ed.* **2016**, *55*, 6004–6007.[6]
Pd/C	0.0027	1.1	

7. Reference

(1) Mao, Z. T.; Rumptz, J. R.; Campbell, C. T. Energetics of Ag Adsorption on and Adhesion to Rutile TiO(100) Studied by Microcalorimetry. *Journal of Physical Chemistry C* **2021**, *125* (5), 3036–3046. DOI: 10.1021/acs.jpcc.0c10504.

(2) Su, L. X.; Zhao, Y. M.; Jin, Y. M.; Liu, Z. Y.; Cui, H. S.; Luo, W. Identifying the Role of Hydroxyl Binding Energy in a Non-Monotonous Behavior of Pd-PdS for Hydrogen Oxidation Reaction. *Adv. Funct. Mater.* **2022**, *32* (27), 2113047. DOI: 211304710.1002/adfm.202113047.

(3) Singh, R. K.; Davydova, E. S.; Douglin, J.; Godoy, A. O.; Tan, H.; Bellini, M.; Allen, B. J.; Jankovic, J.; Miller, H. A.; Alba-Rubio, A. C.; et al. Synthesis of CeOx-Decorated Pd/C Catalysts by Controlled Surface Reactions for Hydrogen Oxidation in Anion Exchange Membrane Fuel Cells. *Adv. Funct. Mater.* **2020**, *30* (38), 2002087. DOI: 10.1002/adfm.202002087.

(4) Miller, H. A.; Vizza, F.; Marelli, M.; Zadick, A.; Dubau, L.; Chatenet, M.; Geiger, S.; Cherevko, S.; Doan, H.; Pavlicek, R. K.; et al. Highly active nanostructured palladium-ceria electrocatalysts for the hydrogen oxidation reaction in alkaline medium. *Nano Energy* **2017**, *33*, 293–305. DOI: 10.1016/j.nanoen.2017.01.051.

(5) Qiu, Y.; Xin, L.; Li, Y.; McCrum, I. T.; Guo, F.; Ma, T.; Ren, Y.; Liu, Q.; Zhou, L.; Gu, S.; et al. BCC-Phased PdCu Alloy as a Highly Active Electrocatalyst for Hydrogen Oxidation in Alkaline Electrolytes. *J. Am. Chem. Soc.* **2018**, *140* (48), 16580–16588. DOI: 10.1021/jacs.8b08356.

(6) Miller, H. A.; Lavacchi, A.; Vizza, F.; Marelli, M.; Di Benedetto, F.; D'Acapito, F.; Paska, Y.; Page, M.; Dekel, D. R. A Pd/C-CeO2 Anode Catalyst for High-Performance Platinum-Free Anion Exchange Membrane Fuel Cells. *Angew. Chem. Int. Ed.* **2016**, *55* (20), 6004–6007. DOI: 10.1002/anie.201600647.
